# Harnessing exosomes for targeted drug delivery systems to combat brain cancer

**DOI:** 10.1186/s12935-025-03731-z

**Published:** 2025-04-15

**Authors:** Abdullah E. Basyoni, Amira Atta, Maha M. Salem, Tarek M. Mohamed

**Affiliations:** Biochemistry Division, Chemistry Department, Faculty of Science, Tanta University, Tanta, 31527 Egypt

**Keywords:** Exosomes, Nanocarriers, Brain cancer, Targeted drug delivery

## Abstract

Brain cancer remains a significant challenge in the field of oncology, primarily because of its aggressive nature and the limited treatment options available. Conventional therapies often fall short in effectively targeting tumor cells, while sparing healthy brain tissue from collateral damage. However, exosomes are now recognized as promising nanocarriers for targeted drug delivery. These naturally occurring extracellular vesicles can cross the blood–brain barrier and selectively interact with cancer cells. Utilizing exosomes as drug delivery vehicles offers a novel approach with significant potential for targeted therapy. By encapsulating therapeutic agents within exosomes, drugs can be specifically targeted to tumor cells, maximizing their impact whilst minimizing damage to healthy brain tissue. Furthermore, exosomes can be modified to display molecules that specifically recognize and bind to cancer cells, further enhancing their precision and efficacy. While exosome-based therapies show potential, scalability, purification, and clinical application challenges remain. The scalability of exosome production, purification, and modification techniques remains a hurdle that must be overcome for clinical translation. Additionally, the intricate interactions between the tumor microenvironment and exosomes necessitate further research to optimize therapeutic outcomes. The review explores applications and future perspectives of exosome-based therapies in advancing targeted brain cancer treatment.

## Overview of brain cancer

**Fig. 1:**
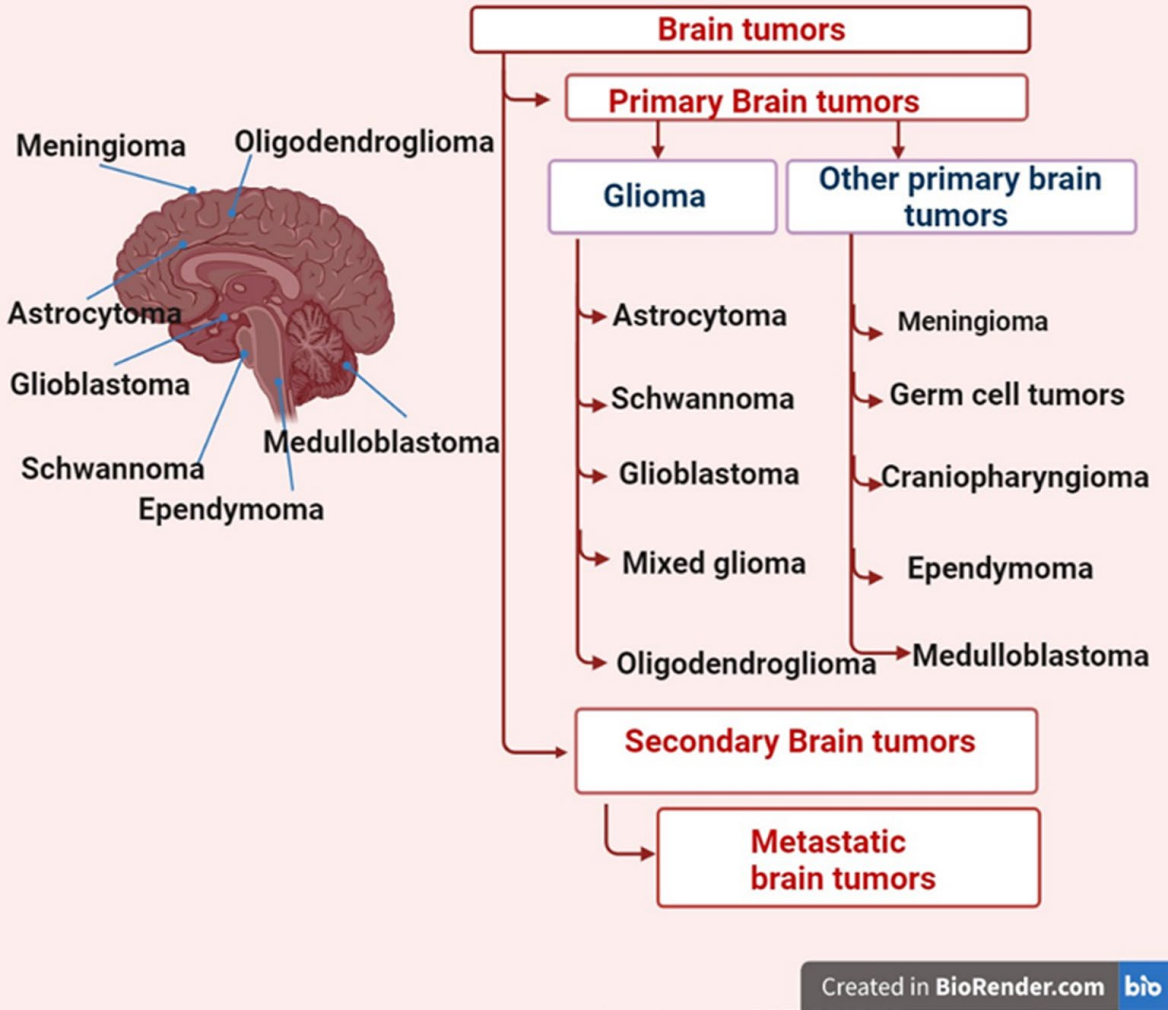
Brain cancer, includes primary brain tumors develop within the actual brain tissue and secondary brain tumors

Brain cancer, a devastating disease that affects the central nervous system, includes primary brain tumors that develop within the actual brain tissue and secondary brain tumors, metastatic tumors originate from cancers in other parts of the body and spread to the brain (Table 1) (Fig. 1) [1]. These tumors typically arise from primary cancers in organs such as the lungs, breasts, or colon, and spread to the brain through the bloodstream or lymphatic system. Brain cancer can be benign or malignant. Gupta Longati, a Russian scientist, made the initial observation of brain cancer in 1873 [2]. He was looking at the brain of a lifeless individual who had allegedly died from a benign tumor. However, he found out that it was indeed a cancerous tumor.

**Table 1:** Summarizing the key characteristics of various brain tumor types

Tumor type	Origin	Characteristics	Key notes
Astrocytoma	Astrocyte cells in the cerebrum	Most common glioma; can range from low-grade to highly malignant forms	Tumor grade determines prognosis and treatment approach
Oligodendroglioma	Oligodendrocytes	Rare, slow-growing tumors; more common in young to middle-aged adults	Uncommon in children; associated with better prognosis
Glioblastoma	Glial cells	Aggressive and fast-growing; divided into primary (de novo) and secondary types	Challenges include poor prognosis and limited treatments
Mixed Glioma	Multiple glial cell types	Tumors arise from combinations of ependymal, astrocyte, and oligodendrocyte cells	Behavior depends on tumor grade
Schwannoma	Schwann cells in peripheral nerves	Typically benign; affects nerve roots or peripheral nerves	Often associated with hearing loss if involving acoustic nerve
Meningioma	Meningeal arachnoid matter	Most common non-glial brain tumor; generally slow-growing	Constitutes 38% of primary brain cancers
Germ Cell Tumors	Germ cells	Rare; primarily occurs in ovaries, testicles, or other locations, including the brain	Can secrete hormones or proteins detectable in blood tests
Craniopharyngioma	Near the pituitary gland	Slow growth; often impacts hormonal balance due to proximity to the pituitary	Two types: papillary and adamantinoma Tous
Medulloblastoma	The cerebellum, near the brainstem	Highly malignant; rapid growth and potential to spread	Commonly seen in children; responsive to radiation therapy
Secondary Brain Cancer	Cancers from other organs (e.g., lung, breast, colon)	Tumors metastasize to the brain via bloodstream or lymphatic system	Often indicates advanced-stage primary cancer

### Primary brain tumors

- Gliomas
  - Astrocytoma: Tumors arising from star-shaped astrocyte cells in the cerebrum.
  - Oligodendroglioma: Tumors originating from oligodendrocytes, more common in adults [3, 4].
  - Glioblastoma: Aggressive tumors classified into primary (de novo) and secondary glioblastomas.
  - Mixed Glioma: Tumors arising from multiple cell types such as ependymal and oligodendrocyte cells. Behavior varies based on tumor grade.
- Schwannoma
  Benign tumors develop from Schwann cells, typically in peripheral nerves.
- Other types
  - Meningioma: Tumors originating from meningeal arachnoid matter, constituting 38% of primary brain cancers.
  - Germ Cell Tumors: Rare tumors arise from germ cells.
  - Craniopharyngioma: Growths near the pituitary gland.
  - Medulloblastoma: Rapidly growing tumors in the cerebellum [5].

### Secondary brain cancer

Secondary (metastatic) brain cancers originate from malignancies in other parts of the body, such as the lungs, breasts, or colon, and spread to the brain [6, 7].

## Risk factors for brain cancer

**Fig. 2:**
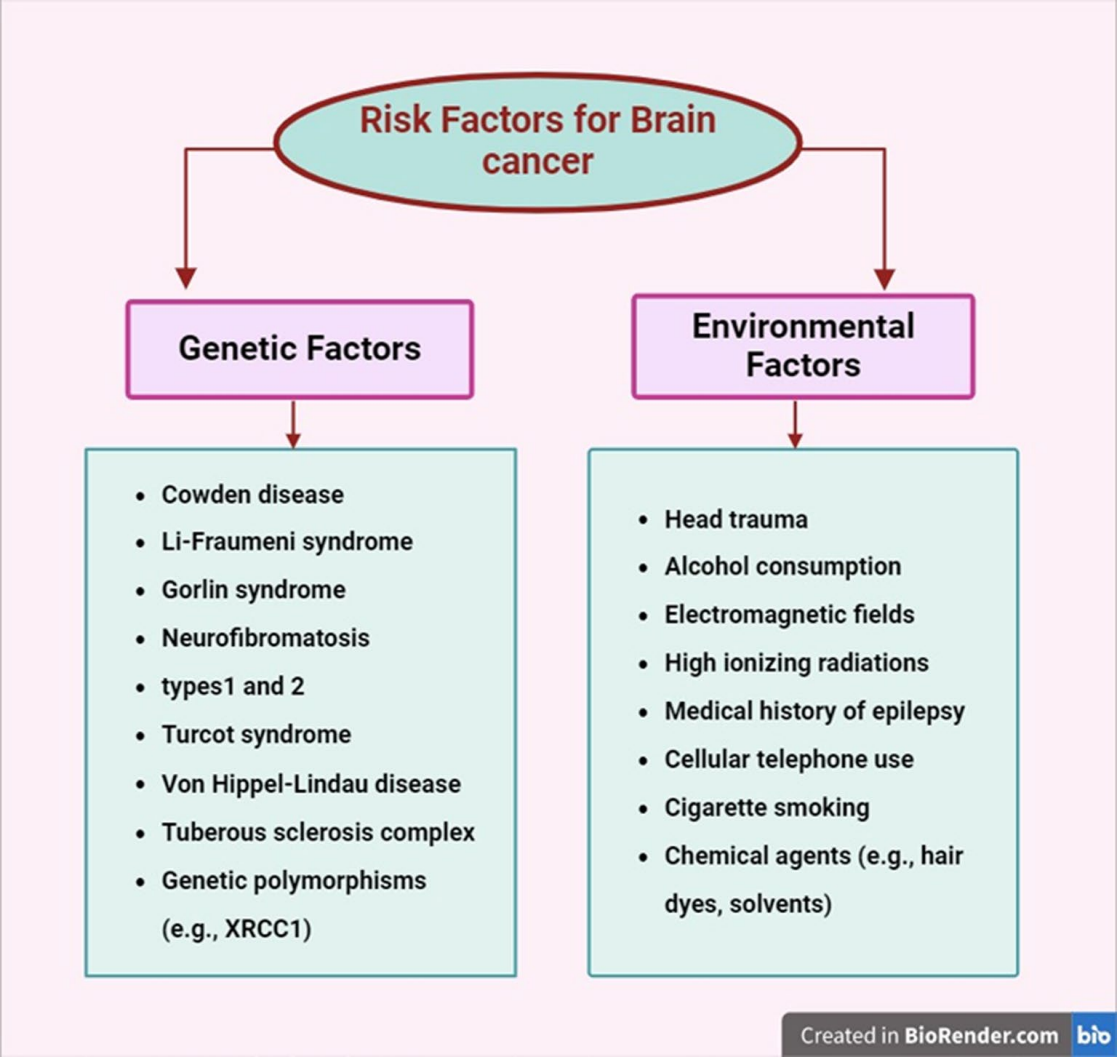
Environmental and genetic risk factors of brain cancer

Brain cancer risk factors can be broadly categorized into environmental and genetic factors (Fig. 2).

### Environmental factors

- Lifestyle choices: Unbalanced diet, smoking, excessive alcohol consumption, and physical inactivity have been implicated in various cancers, including brain cancer, through indirect mechanisms such as increased inflammation and oxidative stress [8, 9].
- Radiation exposure: High levels of ionizing radiation, such as from medical imaging or occupational exposure, significantly increase brain cancer risk.
- Chemical exposure: Carcinogenic substances in industrial chemicals and pollutants are well-documented contributors to cancer risk [10].
- Dietary factors: Consumption of nitrite-preserved foods has been linked to an increased risk of certain brain tumors due to the potential for nitrosamine formation [11].

### Genetic factors

- Inherited mutations: Genetic predispositions, such as mutations in tumor protein 53 (TP53), Phosphatase and TENsin homolog (PTEN), or Neurofibromatosis type 1 (NF1) genes, are associated with an increased risk of gliomas and other brain tumors [11].
- Familial syndromes: Conditions like Li-Fraumeni syndrome and neurofibromatosis are linked to higher brain cancer incidence (Fig. 2).

While certain medications, such as immunosuppressive drugs used in transplant patients, have been associated with an increased risk of specific cancers, robust evidence linking common medications (e.g., over-the-counter drugs or sleeping pills) to brain cancer is lacking. Any mention of such associations has been removed to maintain scientific accuracy [10, 12].

## Mechanism of metastatic brain cancer

**Fig. 3:**
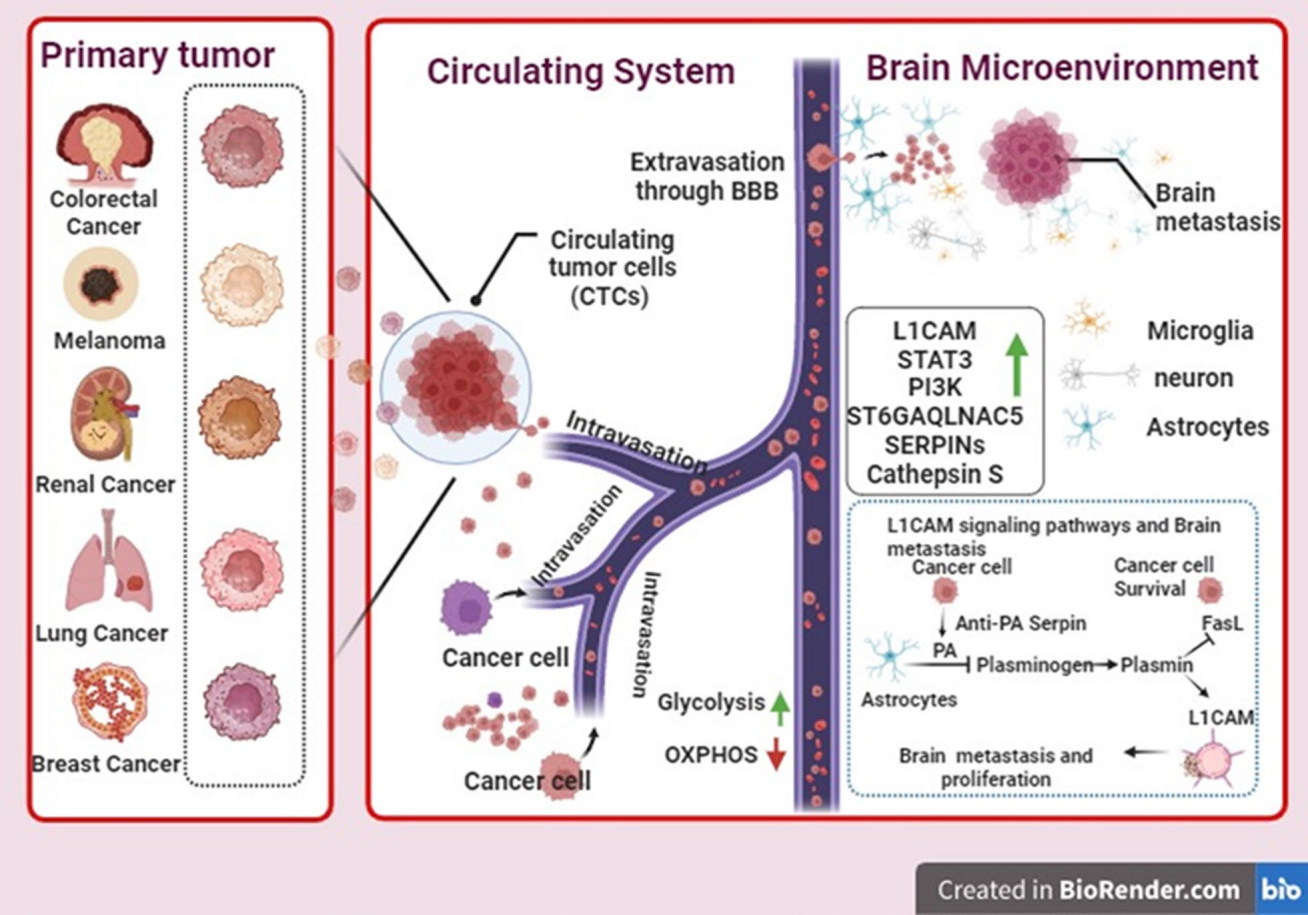
The mechanism of brain metastasis involves the release of Circulating tumor cells (CTCs) from the primary tumor into the bloodstream, extravasation into the brain tissue through the blood–brain barrier (BBB), and adaptation to the brain microenvironment. [L1cell adhesion molecule (L1CAM), Signal transducer and activator of transcription 3 (STAT3), Fas ligand (FasL), phosphatidylinositol-3 kinase (PI3K), Plasminogen activator (PA), the serine proteinase inhibitor (Serpin), Oxidative phosphorylation (OXPHOS)]

The intricate process of cancer cells spreading from a primary tumor to the brain is known as brain metastasis. Circulating tumor cells (CTCs) from the primary tumor that survive in the bloodstream and go to the brain is the major mechanism of brain metastasis from primary cancers (Fig. 3) [13]. Once within the brain, these cells can go dormant and avoid immune system recognition. They can also facilitate their migration in the brain by activating signaling pathways such as cathepsin S, L1 cell adhesion molecule (L1CAM), and Signal transducer and activator of transcription 3 (STAT3) [13, 14]. Furthermore, they can prevent the activation of the protein Fas ligand (FasL), which causes cancer cells to undergo apoptosis and spread to the brain [15].

When CTCs are released into the bloodstream from the main tumor, brain metastasis starts. In circulation, these cells are subject to shear pressures and immunological monitoring, and they must survive. CTCs can attach to the brain’s endothelium of blood arteries after they are in the bloodstream. This is made possible via interactions between the endothelial cells and adhesion molecules on the CTCs. The CTCs can extravasate and enter the brain tissue because of this adhesion.

Upon entering the brain, CTCs face the challenge of surviving and proliferating in a foreign microenvironment [13, 16]. The cellular and molecular makeup of the brain microenvironment sets it apart from those of other organs. CTCs need to adjust to this setting to spread throughout the brain. One way they achieve this is by entering a dormant state, where they temporarily cease proliferation and evade immune detection. This dormancy allows the CTCs to evade therapeutic interventions and remain undetected for extended periods.

CTCs use a variety of signaling pathways in addition to dormancy to encourage their migration and proliferation within the brain. One such pathway that aids in cell invasion, proliferation, and survival is the signal transducer and activator of the STAT3 pathway. To improve their survival and proliferation in the brain microenvironment, CTCs can stimulate STAT3 signaling [17]. Another important pathway is L1CAM, which is implicated in promoting cell motility and invasion. CTCs can upregulate L1CAM to facilitate their migration within the brain tissue [18], contributing to the formation of metastatic lesions.

Furthermore, CTCs can exploit the protease cathepsin S to facilitate their migration and invasion in the brain [13]. Cathepsin S can encourage CTC migration and invasion in the brain and is involved in modifying the extracellular matrix. Through the cleavage of blood–brain barrier (BBB) junctional adhesion molecules (JAMs), such as JAM-B [11], cathepsin S facilitates the migration of CTCs into the brain tissue, contributing to the formation of brain metastases.

Moreover, CTCs can inhibit the activation of FasL, a protein that induces apoptosis in cancer cells [19]. Essentially, the process of brain metastasis entails the discharge of CTCs from the original tumor into the bloodstream, their endurance and attachment within the brain microvasculature, their infiltration into the brain tissue, their adjustment to the brain microenvironment via signaling pathway activation and dormancy, and the suppression of apoptosis to generate brain metastases. Comprehending the complex processes associated with brain metastasis is essential for creating tailored treatments to avoid or manage this severe cancer consequence [20].

## Treatments for brain cancer

The two main goals of cancer treatment options are to either remove the tumor or, by decreasing the tumor, provide relief from the symptoms of cancer. The kind, location, and size of the tumor, as well as the patient’s age, medical history, overall health, and types of symptoms, all influence the treatment plan that is selected. The several approaches used to treat brain cancer [21] include.

### Surgery

The major treatment technique in the early stages of a benign tumor is surgical excision of the tumor while maintaining brain function. In most cases, surgery is the only treatment available for low-grade tumors. Cancer symptoms are lessened by surgically removing the tumor, which also decreases intracranial pressure caused by the tumor. With few or no side effects [22], it is the simplest and safest method of treating brain cancer. Surgery can only be used as a therapeutic option if the tumor is at a place that may be reached without endangering vital brain functions. This is a limitation of the procedure. Headache, weakness, fatigue, brain swelling, or a build-up of fluid in the brain are common post-operative side effects. Brain injury can also be a very significant issue. Epilepsy surgery can also result in cognitive, verbal, and visual impairments [9].

### Radiation

Brain cancer shrinks as a result of radiation therapy, which uses gamma, x-, and proton beams to kill or destroy malignant cells. Typically, radiation therapy is administered five days a week for 6 weeks [23]. In general, there are two forms of radiation therapy: exterior radiation treatment, including whole brain radiation therapy (WBR), which delivers a lower radiation dose after each treatment, and stereo static radiosurgery (SRS), which delivers a high dose of radiation in a single treatment. Conversely, brachytherapy, or implant-based radiation therapy, is a component of internal radiation therapy. Radiation therapy side effects can include headaches, nausea, edema, exhaustion, and changes in movement or feeling [21].

### Chemotherapy

Chemotherapeutic medications are used in this cancer therapy approach. The main ways that therapeutic drugs work are by preventing blood flow to tumor cells or by interfering with the process of cell division, which kills aberrant cells and causes tumors to shrink [22, 24]. This therapeutic approach’s drawback is that it could harm healthy tissues or cells as well. Chemotherapeutic treatment commonly causes side effects including exhaustion, thirst, weakness, nausea, and decreased white blood cell counts, all of which raise the risk of infection.

### Hormonal therapy

Hormonal therapy is a type of cancer treatment in which the growth of the cancer is slowed or stopped by hormones. It is a non-toxic treatment for prostate and estrogen receptor-positive breast cancers. The two primary approaches to hormonal therapy are blocking the body’s ability to produce hormones and interfering with the hormones’ ability to function in the body [24, 25].

### Photodynamic therapy

A photosensitizer is used in photodynamic therapy to kill the malignancy. A photosensitizer is a type of photosensitive material that, when it absorbs light with a certain wavelength that is concentrated on the target cell, starts a photochemical or photophysical response [26]. The photosensitizer converts light energy into molecular oxygen upon absorption, resulting in the formation of reactive oxygen species (ROS), which include free radicals and singlet oxygen. Cellular toxicity is caused by the harmful effects of ROS.

### Immunotherapy

Biotherapy known as immunotherapy works by sensitizing the immune system to cancer, hence reducing side effects and increasing selectivity. Malignancy growth and metastasis can use immunosuppressive mechanisms to prevent the immune system from recognizing the malignancy. Cancer immunotherapy aims to strengthen the immune system to fight these malignant cells. In cancer immunotherapy, there are four main approaches: immunomodulation, targeted antibodies, adoptive cell treatment, and cancer vaccines. Priming the host immune system enhances the immunological response through immunomodulation. Essentially [27], it works by stimulating T cells to kill more tumor cells through the presentation of antigens to T cells. Although there are other drugs available, cytokines are the most popular ones for immunomodulation. By modifying the patient’s T cells in a lab to target the cancer cells more effectively, adoptive cell therapy is an immunotherapy technique used to support the immune system’s defense against cancer cells. One form of active immunotherapy that aims to stimulate the immune system’s effector activities is the cancer vaccine. For the immune system to develop memory cells resistant to these antigens, cancer vaccines are responsible for exposing the immune system to particular antigens produced on the surface of cancer cells [28, 29].

### Targeted cancer therapy

The delivery of medications to genes or proteins unique to cancer cells or the tissue milieu that fosters the growth of cancer is known as targeted treatment. Targeted therapy includes developing drugs that stop cancer cells from growing, aid in the regulation of the cell cycle, cause apoptosis or autophagy, or target specific cancer cells with toxic compounds in an attempt to kill them [30]. Oral tiny medicines or monoclonal antibodies are used in targeted therapy. Cancer cells’ surface proteins and receptors are bound by monoclonal antibodies. By blocking substances that signal angiogenesis, or the growth of cancer cells, these large molecules prevent the spread of cancer. Small-molecule drugs are another type of targeted therapy; because of their low molecular weight, they can penetrate the cell surface and either stop the growth of tumor cells or kill them [31].

### Challenges in targeting therapy for brain cancer

The most difficult obstacle to the effective transport of medicinal medicines into the brain is the blood–brain barrier (BBB). It functions as a physiological and physical barrier to the transfer of medications to the brain. The capillary endothelial cells’ tight connections are what give the blood–brain barrier its impermeability. According to reports, the majority of small molecular-weight medicinal compounds and nearly all high molecular-weight therapeutic drugs lack the innate ability to pass the blood–brain barrier. For a drug ingredient or formulation to penetrate the blood–brain barrier [32], it is ideal if it has a tiny molecular weight (500 Da), is lipid-soluble, log P < 5, has electrically neutral molecules, and weak bases that can diffuse passively across the barrier. Efflux transporters are engaged in the transfer of solutes out of the brain endothelial cells and are additional barriers to the delivery of medicine throughout the brain. The drug substance that has entered the brain is removed by the efflux transporters. A feeble Enhanced Permeability and Retention (EPR) effect is a further obstacle to the treatment of brain tumors [33]. By adding lipophilic or amphiphilic groups to the blood–brain barrier at specific points, it is possible to increase drug delivery to the brain while preserving the equilibrium between hydrophilic and lipophilic conjugates.

## Conventional nanocarriers

Conventional nanocarriers can be broadly classified into two groups as shown in Tables 2 and 3. Liposomes and micelles are examples of organic nanocarriers, On the other hand, inorganic nanocarriers include layered double hydroxides, graphene, nanoparticles that are magnetic, nanoparticles of gold, mesoporous silicon dioxide nanoparticles, and quantum dots. Exosomes possess numerous benefits over traditional nanocarriers and hold great promise for the treatment of tumors in clinical settings. First off, exosomes’ phospholipid bilayer shape allowed them to deliver medications steadily, avoiding the breakdown of drugs by enzymes and prolonging their half-lives. Additionally, their membranes interacted well with the target cells. As a result, the loaded drug’s bioavailability also increased. The immunogenicity and toxicity of exosomes were also inferior to those of liposomes, a conventional drug carrier. Furthermore, we can not disregard their small frame. Exosomes can move throughout tumor tissue and extravasate in tumor vasculature to treat malignancies because of their nano size. Exosomes can therefore also pass across some physiological barriers, such as the blood–brain barrier. An additional benefit is that the medication’s potency is increased when it is placed into exosomes.

**Table 2:** Inorganic nanocarriers

Nanocarrier	Advantages	Disadvantages
Mesoporous Silica	Highly stable; easily functionalized for targeted delivery (e.g., across the blood–brain barrier)	Limited biocompatibility without surface modification; potential for toxicity
Magnetic Nanoparticles	Excellent biocompatibility; enable magnetic field-based targeting; effective for hyperthermia treatments	Size and heating control during hyperthermia are challenging; toxicity concerns
Gold Nanoparticles	High electron density; easy conjugation with biomolecules; surface-enhanced Raman scattering capabilities	Expensive synthesis; potential biosafety concerns; limited scalability
Carbon Nanotubes	High drug-loading capacity; superior transmembrane capabilities	Cytotoxicity; poor dispersibility; surface functionalization challenges

**Table 3:** Organic nanocarriers

Nanocarrier	Advantages	Disadvantages
Liposomes	Biodegradable; biocompatible; can encapsulate hydrophilic, hydrophobic, and amphiphilic drugs	Low stability; prone to oxidation; challenging sterilization
Micelles	High drug-loading capacity for hydrophobic drugs; good stability in biological fluids	Restricted to hydrophobic drugs; limited application for hydrophilic molecules
Dendrimers	High surface functionality; monodisperse size; adaptable internal cavities	High synthesis cost; complex purification; scalability issues
Exosomes	Low immunogenicity; high bioavailability; effective for crossing the blood–brain barrier	Difficult large-scale production; challenges in isolation and purification

## Exosomes

A class of naturally occurring nanoscale membrane vesicles called exosomes is produced by live cells via a sequence of regulatory mechanisms called “endocytosis-fusion-efflux”. To put it succinctly, exosomes were initially identified about 40 years ago. The understanding of exosomes has substantially increased in the last few decades. Exosomes were first believed to be a route for cell excretion, but more investigation showed that they are also a medium involved in material and information transfer between cells, transporting host cell proteins, lipids, nucleic acids, and other materials. As a result, exosomes are employed as a type of nanocarrier to transfer medications like paclitaxel or nucleic acids to cure a variety of illnesses including cancers [34–36]. Exosome utilization and production of minimal or harmless granules having high-efficiency exosome loading, which were used in the treatment of cancer, has quietly emerged as a hot topic in the study. To serve as a guide for cancer treatment in the future, this paper defines exosomes in detail and provides an overview of their use as nanocarrier-loaded medications in tumor therapy.

## Origin and structure

**Fig. 4:**
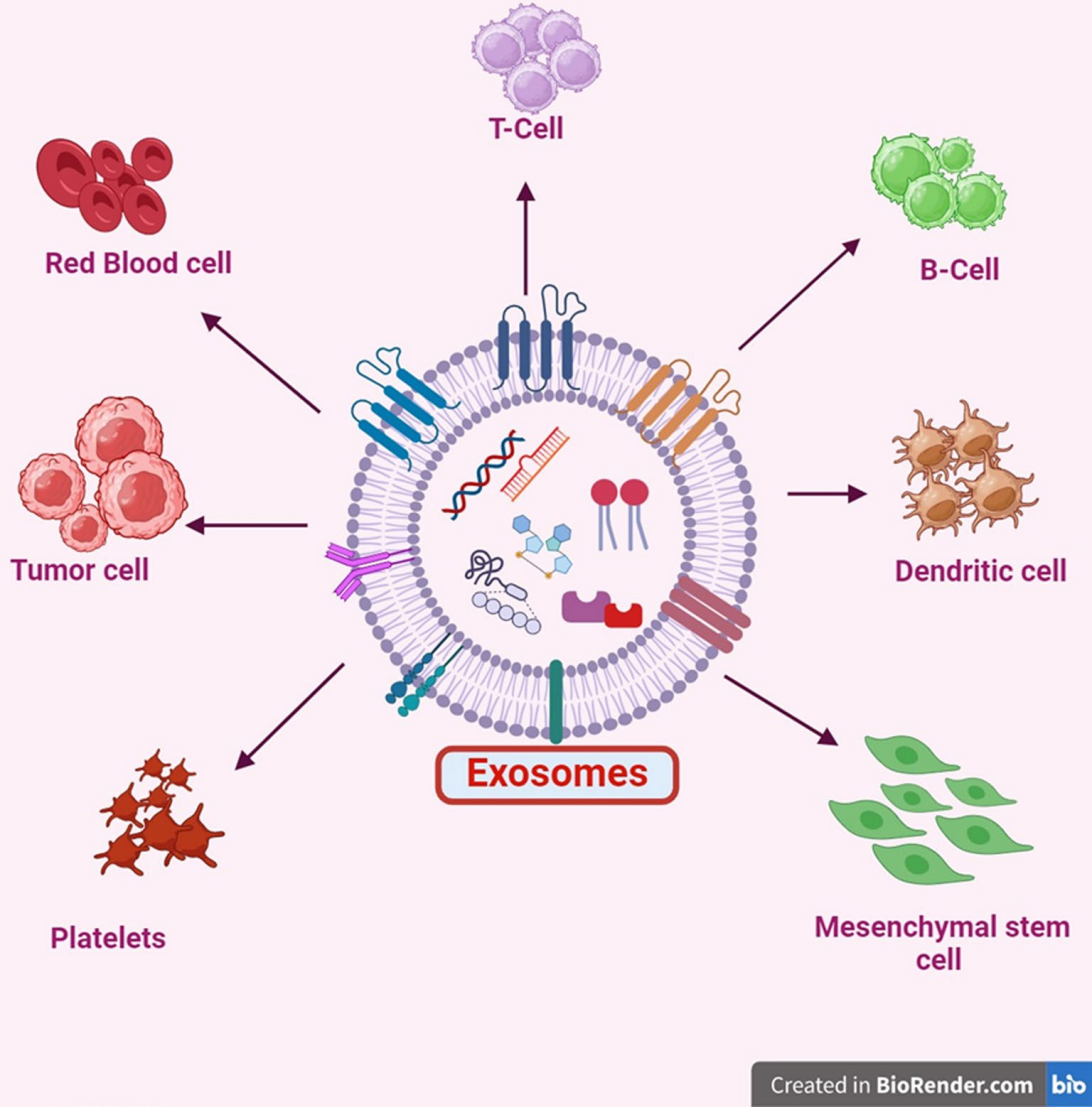
Exosome secretion by various cells

Exosomes are a kind of lipid bilayer vesicles that have a round shape and a diameter that ranges from 30 to 150 nm. Their monodisperse distribution and round shape are seen in the electron microscope image of exosomes [37]. In 1981, Trams et al. discovered that exosomes exist. By using electron microscopy, Pan et al. described the creation of exosomes in 1985. The term “exosome” was not formally introduced until 1987, by Johnstone et al. Exosomes are composed of various components. They are typically secreted by nearly all mammalian cells (Fig. 4), including tumor cells, mesenchymal stem cells, epithelial cells, endothelial cells, dendritic cells, and T and B lymphocytes, and operate as messengers and transmitters in cellular crosstalk [38].

### Exosome biogenesis

**Fig. 5:**
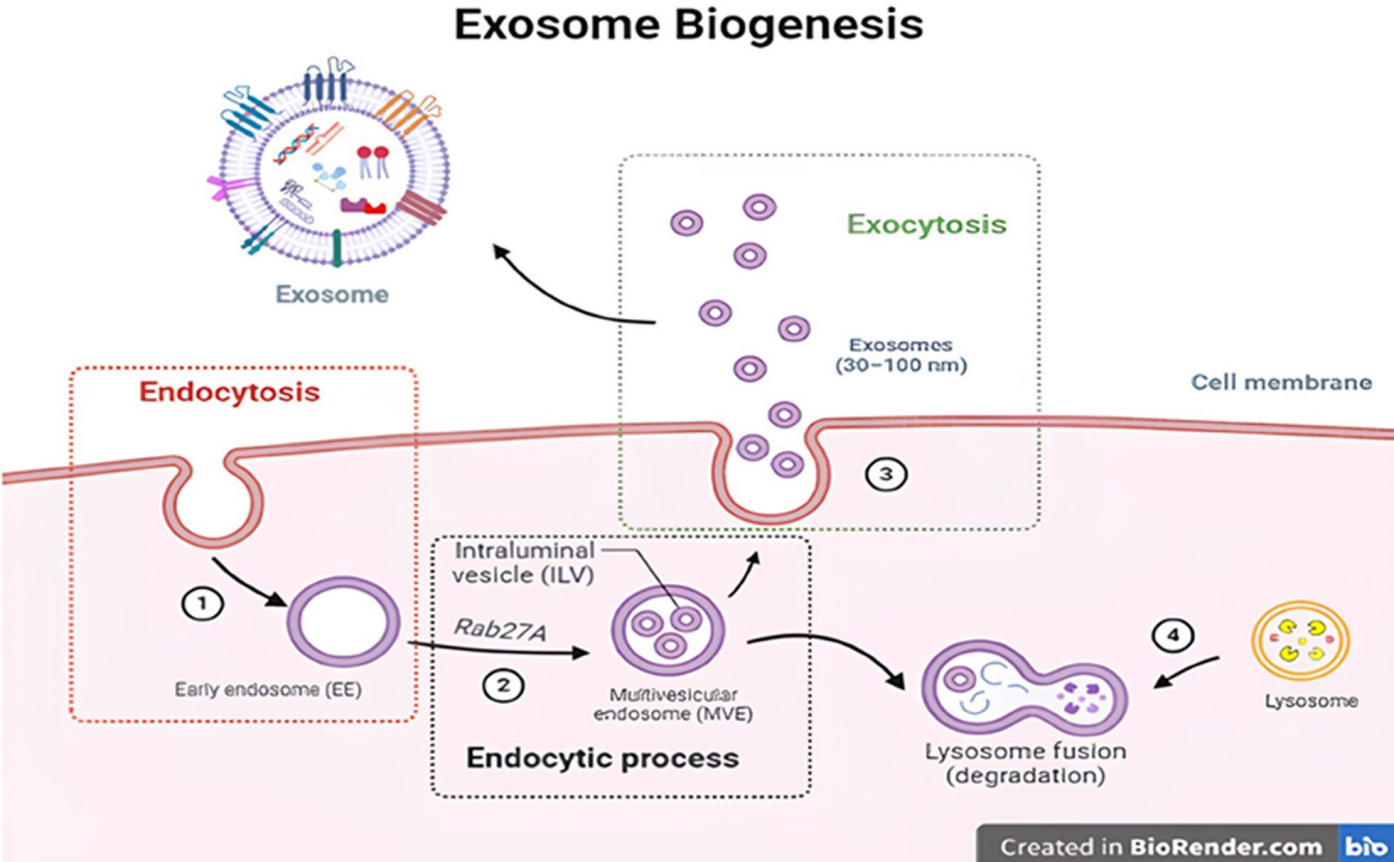
The endocytic process, the endosome process, and exocytosis are the three phases of the exosome process. When endocytosis occurs, the endosome gets tapped and matures into an older endosome that has numerous intraluminal vesicles (ILVs), or multi-vesicle bodies (MVBs). Exosomes represent the tiny vesicles that MVBs discharge into the external membranes

Exosomes were generated by the endocytic pathway. According to Fig. 5, the particular generation process is as follows: In the early endosomal (EE) stage, endocytosis forms early endosomes in the cell membrane; in the late endosomal stage, Endosomal Sorting Complex Required Transport (ESCRT-0) binds to the early endometrial membrane’s specific receptors via the ubiquitination binding site, and selectively splices a section of the cytoplasm, causing ESCRT-I to attach to ESCRT-0, which in turn allows ESCRT-II to attach to ESCRT-I, forming intraluminal particles through budding. Afterward, ESCRT-III chops the neck bud, and ESCRT-I and ESCRT-II collaborate to promote the formation of intraluminal vesicles (ILVs) [39]. As a result of the ILVs’ separation from the endosomal membrane, other substances are released into the endosomal cavity. Additionally, the budding process is completed, resulting in the formation of mature late endosomes, which are also known as multi-vesicle bodies (MVBs) because they contain multiple ILVs. Exocytosis: after that, some MVBs are broken down by fusing with lysosomes, while some of them fuse with the plasma membrane, releasing the MVBs’ intracellular vesicles into the extracellular media in the form of exosomes. In addition, some MVBs are paired with the Golgi apparatus to recycle [40].

### Exosome composition

**Fig. 6:**
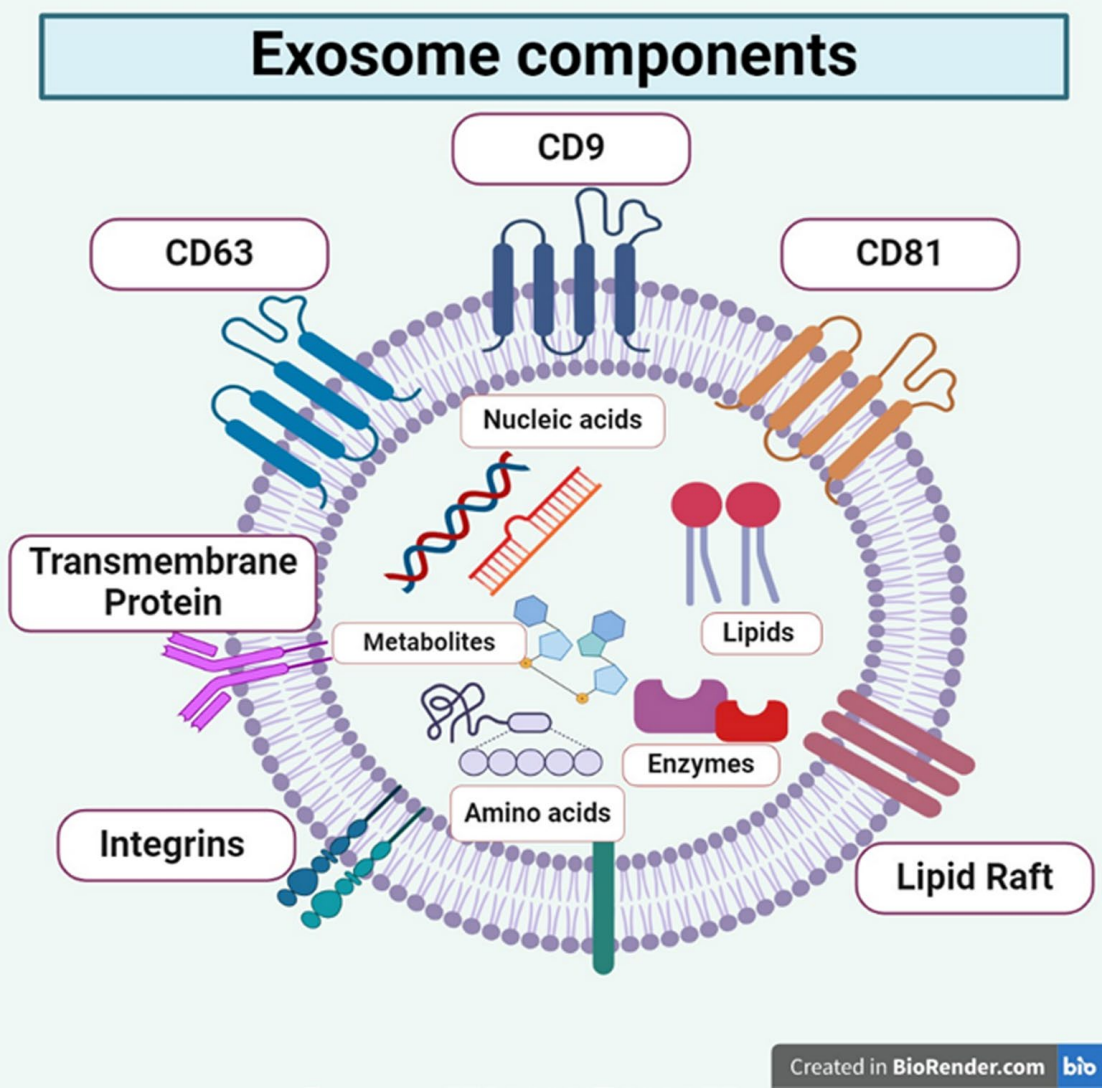
Exosome components with lipids, integrins, amino acids, nucleic acid, and metabolites

The size and cargo of exosomes might vary even when they originate from the same cell. Exosomes originating from different sources do share some payloads, though. Several investigations on exosomes have reported the presence of a wide range of biomolecules, including proteins, lipids, RNAs, and DNAs (Fig. 6). The majority of lipids found in exosomes, including cholesterol, sphingomyelin, and phosphatidylserine, are recognized to be elements of plasma membranes. Heat shock proteins (HSP70, HSP90), integrins myosin heavy chain (MHC) class II proteins, proteins such as (Rab GTPases, annexins), etc. are among the proteins found in exosomes [41]. Furthermore, exosomes have proteins transcribed on their outer layer, where they interact with recipient cell surface receptors to trigger intracellular signaling. Exosomes have been found to include all RNA species, including transfer RNA, long noncoding RNA, messenger RNA (mRNA), and microRNA (miRNA). Certain RNAs are reportedly actively, rather than passively, sorted and transported into exosomes, where they eventually affect host cells’ transcripts, according to numerous studies. Remarkably, Batagov et al. also discovered that a particular RNA fragment might allow the 3′-untranslated region (UTR) of mRNA to reach the exosome. Furthermore, single-stranded DNA was found in exosomes by Balaj et al., while whole genome sequencing by Kalluri and colleagues demonstrated the presence of double-stranded genomic DNA pieces of at least 10 kb in exosomes [42].

## Intercellular communication mediated by exosomes

### Uptake of exosomes

Cosseti et al. discovered that in brain stem/precursor cells, interferon (IFN) binds to IFN-receptor 1 to form a complex that then triggers signaling transmission through this complex. Furthermore, research has indicated that exosomes may fuse with recipient cell membranes to deliver their contents to target cells [43], while the precise molecular process is yet unknown. Many studies conducted recently indicate that the primary mechanism of exosome uptake is internalization by endocytic pathways, including phagocytosis, caveolin-mediated endocytosis, macropinocytosis, clathrin-mediated endocytosis, and lipid raft-mediated endocytosis. The result is cell-specificity due to surface chemicals interacting with particular cells.

### Tumor microenvironment

**Fig. 7:**
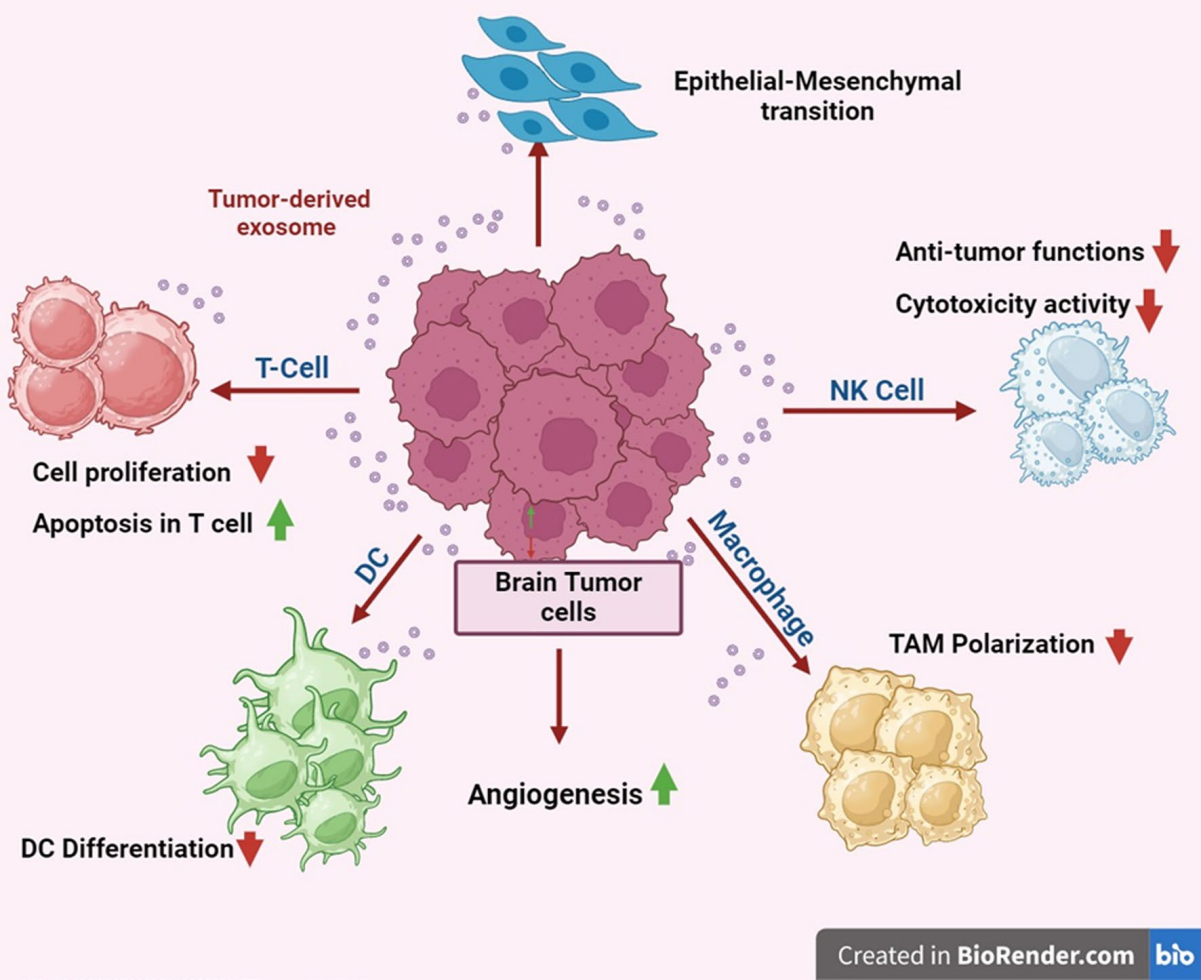
Intercellular communication mediated by exosomes. [Dendritic cells (DCs), Natural-killer (NK), and Tumor-associated macrophages (TAMs)]

Exosomes transport chemicals and are essential for intercellular communication. Biomolecules produced from exosomes secreted by donor cells have the potential to significantly alter recipient cells’ biological response. Zhang et al. discovered that the human monocytic leukemia cell line (THP-1) secretes a sizable amount of miRNA-150, which may facilitate target cell migration by suppressing target gene expression. In a different investigation, Wang et al. showed that the administration of transient receptor potential polycystic 2 (TRPP2) siRNA via exosomes dramatically suppresses the expression of TRPP2 and the epithelial-mesenchymal transition in FaDu cells. Additionally, several investigations demonstrated that exosomes with functional proteins can facilitate cell-to-cell contact and exert significant influence over the target cell’s signaling pathway, which is linked to the advancement of cancer. Consequently, exosome-mediated cell-to-cell contact is a useful method for delivering various cellular macromolecules and influencing target cell phenotypes and activities [44]. Natural-killer (NK) cells play a crucial role in immune surveillance and act as the primary defense in controlling the growth and metastasis of cancer in our bodies. Through the delivery of killer proteins along with characteristic NK and exosome signals, NK cell-derived exosomes confer lethal action to tumor cells. Like NK cells, macrophages produce pro-inflammatory cytokines like interleukin 6 (IL-6) and tumor necrosis factor-alpha (TNF-α), as well as reactive oxygen/nitrogen species, which are crucial for both innate host defense and tumor cell death. Tumor-associated macrophages (TAMs) in the surroundings of the tumor have been linked to the display of an M2-like phenotype. Macrophages are generally classed into M1 or M2 kinds. These TAM-derived exosomes mediate cell-to-cell contact with other immune cells to induce immune suppression for tumor development. Malignant cells generate tumor-derived exosomes (TEXs), which modify macrophage physiology, inhibit NK cell activity, and promote tumor growth by controlling T cell function (Fig. 7) [43]. Through their ability to control the tumor microenvironment (TME), TEXs are also crucial in the spread of tumors. Exosomes generated from breast cancer cells, for instance, carry miRNAs and oncogenic proteins like miRNA-130a and miRNA-328 that promote tumor growth and spread. Exosomes produced from human pancreatic cells play a crucial role in immune-suppressive processes by promoting metastasis. In a similar vein, Wang and colleagues discovered that TEXs in the gastric tumor cell microenvironment promote the development of primary tumor growth by eliciting a population of immunosuppressive PD1+ tumor-associated macrophages that impede the function of CD8+ T cells Numerous accounts have also been published regarding the investigation of TEXs’ role in angiogenesis [42]. The primary indicator of the advancement of a tumor is angiogenesis, which provides the blood vessels linked to the tumor and serves as the target of cancer immunotherapy. TEXs play a role in the induction of new arteries during the early stages of cancer development in a variety of tumor forms, such as hypoxic lung cancer, malignant mesothelioma, and glioblastoma [5]. Furthermore, because of changes in tumor metabolism, there are ten times more of these TEXs than exosomes generated from healthy cells. Consequently, because of their role in TME. Numerous recent research have examined the function of TEXs as a biomarker [45]. In a different study, Mousavi et al. looked into the possibility that TEXs regulating the development of cancer could serve as possible biomarkers for colorectal cancer. These results show that TEXs have a variety of effects on the pathophysiology of cancer. An overview of exosomes produced from tumors and their function in the tumor microenvironment. Cancer cells can produce exosomes, which can stimulate the development of mesenchymal stem cells (MSCs) and cause other cancer cells to undergo the epithelial-mesenchymal transition [45]. To polarize macrophages toward TAM, a phenotype that supports tumors, cancer cells can stimulate angiogenesis. Exosomes generated from tumors can inhibit immune cells, including dendritic cells (DCs), T cells, and NK cells, from responding to tumors.

## Microenvironment and therapeutic exosome interactions

The interactions between exosomes and the tumor microenvironment are paramount to understanding how these interactions can influence exosome functionality and therapeutic outcomes. The complexity of exosome absorption, involving various proteins and cellular processes like clathrin-dependent endocytosis and macropinocytosis, highlights the intricate mechanisms at play when exosomes interface with recipient cells. Numerous exosomal proteins interact with receptors on the receiving cells to enable exosome absorption [46]. Cell uptake of exosomes may be enhanced by radiotherapy-like modulation of CD29/CD81 complex formation. It has been proposed that using a CD9 antibody against tetraspanin to prevent DCs from absorbing exosomes works just as well. To regulate exosome binding and absorption, integrins (integrins v and 3, i.e., CD51 and CD61), proteoglycans (heparan sulfate proteoglycans), and lectins (C-type lectins DEC-205, galectin-5) collaborate. We still do not fully understand the mechanisms behind exosome-cell interactions, even though an increasing number of protein interactions are known to influence exosome attachment and absorption. The process by which cells absorb molecules through clathrin-coated vesicles in response to certain membrane receptors and their ligands is known as clathrin-dependent endocytosis. As vesicle buds develop and expel their clathrin-coated blisters, the plasma membrane may become distorted. After clathrin breakdown, the contents of a vesicle merged with an endosome were released chlorpromazine may impede this enzymatic pathway [47, 48]. Clathrin-dependent endocytosis is promoted by increases in membrane fusion, membrane curvature, and membrane fission brought on by the GTPase dynamin2. Unlike dynamin-positive cells, dynamin-negative cells dramatically suppress the exosome internalization process. Therefore, dynamin inhibitor therapy or a reduction in caveolin-1 significantly reduces exosome internalization. On the other side, the mechanism by which vesicles ranging in diameter from 0.2 to 10 nm take up external fluids and solutes is known as macropinocytosis. This often occurs in the plasma membrane’s most tightly wrapped regions. Cells employ the macropinocytosis process to absorb certain exosomes. Micropinocytosis is the mechanism by which oligodendrocyte-produced exosomes are delivered to microglia [49]. To ascertain if exosomes are internalized for intercellular communication or removed by phagocytosis, more investigation is required. At last, it is important to mention that exosomes aid in organotrophic metastasis, and other crucial oncogenic signals also contribute to balancing the functioning and selectivity of exosome payloads engaged in this process [50].

## Exosome types

The field of research on the creation of effective drug delivery systems (DDS) has expanded rapidly, and exosomes that have a high rate of bioabsorption and minimal immunogenicity are being targeted as powerful drug carriers. To enable the drug to cross biological barriers (such as cell membranes [39, 41], efflux transporters, and metabolic enzymes) and having high bioavailability in target regions is the primary goal of the DDS. Depending on the kind of exosome source, different physicochemical characteristics may influence the pharmacokinetics of the material [51]. As a result, it’s critical to research how exosomes’ diverse biochemical characteristics originate from distinct sources. This section will be devoted to classifying the many exosome kinds that come from various sources [52].

### Cell-secreted exosomes

Exosomes are nanoscale in size and can be discharged by nearly any kind of cell. Since extracellular vesicles (EVs) vary widely in composition and function. Characterizing exosomes recovered from conditioned cell culture media is relatively well-established in the present EV field, as opposed to exosomes derived from complicated biological fluids like plasma [53]. Differential centrifugation is the known “gold standard” for isolating exosomes as a subset of EVs. Moreover, several techniques are frequently employed in exosome applications, including immunoaffinity chromatography, polymer precipitation, and ultrafiltration. Exosomes from human embryonic kidney (HEK) cells, cancer cells, immune cells, and stem cells have been extracted by numerous research groups; these exosomes differ depending on where they come from [54].

### (HEK)-derived exosomes

Because of its many benefits, including easy growth, low care requirements, and high transfection efficiency, the HEK cell line (HEK293T) is the most often applied in the biopharmaceutical production industry. Exosomes isolated from HEK293T contain membrane similarities to a variety of human tissues, including the epithelium, lung, muscle, lymph, and hepatocytes, according to some prior investigations. This implies that medication distribution to diverse target organs is made possible by HEK-derived exosomes. Furthermore, Zhu et al. found that giving mice repeated doses of HEK293T exosomes for three weeks did not significantly alter the mice’s immune system or cause harm. Apart from its safety-related characteristics, HEK-derived exosomes have the potential to enhance medication delivery and therapeutic efficacy by supplying cancer cells with membrane proteins. Kim et al. [54] used HEK exosomes that had been genetically altered into “xenogeneic” tumor cells. The authors generated minimal vesicular stomatitis virus glycoprotein (mVSVG)-engineered Exosomes (mVSVG-Exo) by transfecting HEK293T cells with plasmids containing mutant vesicular stomatitis virus glycoprotein (mVSVG). The phagocytosis of xenogenized cancer cells by bone marrow-derived macrophages (BMDMs) and bone marrow-derived dendritic cells (BMDCs) was enhanced by mVSVG-Exo. Furthermore, it was discovered that exosomes expressing therapeutic membrane proteins could enhance tumor penetration and antitumor activity. The fundamental component of the tumor microenvironment, the tumor extracellular matrix (ECM), is degraded by native PH20 hyaluronidase-expressing exosomes derived from HEK293T cells, which inhibits tumor growth. These findings are demonstrated in experimental studies by Hong et al. Furthermore, in the tumor-bearing mice model, co-administration of doxorubicin (Dox) and PH20 demonstrated significantly higher anticancer effects in comparison to Dox-only delivery groups [55].

### Cancer cell-derived exosomes

Cancer cells are also thought to be effective exosome makers such as subtypes of Ras-associated binding (Rab) proteins (Rab27a and Rab27b), which are involved in the exosome release mechanism. A trait that sets them is their affinity for their parent cells. Exosomes of tumor cell lines HT1080, and HeLa, a cervical cancer cell line were extracted by Qiao and colleagues, who also found that HT1080 exosomes exhibited twice as much absorption in HT1080 cells as HeLa exosomes. In addition, the researchers used HT1080 exosomes that were drug-loaded with anticancer drugs to perform an in vivo efficacy test [56]. They discovered that this resulted in a considerably higher concentration of HT1080 exosomes at the HT1080 tumor location when compared to HeLa exosomes. Exosomes produced from cancer cells have demonstrated encouraging potential as drug delivery systems; nevertheless, there are still several issues that need to be resolved before using them to treat cancer. First, the pharmacokinetic profile of naive exosomes produced by cancer cells is not optimal. Second, while employing cancer exosomes as medication carriers, it is important to carefully evaluate any potential side effects, as numerous studies have suggested that cancer exosomes may play a role in tumor spreading. Exosomes obtained from cancer patients should be a useful tool for treating the disease if the flaws mentioned above are fixed [57–60].

### Immune cell-derived exosomes

T cell-mediated immunotherapy frequently uses dendritic cells (DCs), which are utilized to convey tumor antigens to naive T cells. However, DCs have a short half-life following activation. Nevertheless, because DC-derived exosomes (DEX) retain the immune stimulation-related capabilities of their source, DEX is suggested as a crucial molecule to supplement the shortcomings of DC-based immunotherapy [61]. First, the exact molecular makeup of each patient’s DEX determines the molecular criteria for biological materials quality control. Second, the DEX surface expresses the ligand peptides that stimulate NK cells. Furthermore, compared to DC which enables DEX therapy to last up to six months with just one leukapheresis. DEX extracted from immature DCs generally displayed a lack of expression of immunostimulatory (e.g., CD86), which can avert unanticipated immunological reactions brought on by the activation of naive T cells [53].

### Stem cell-derived exosomes

One of the cell types known to secrete exosomes is also thought to be a perfect source to manufacture exosomes for therapeutic application because they can be isolated from a range of human tissues and have a high growth potential. The Kalluri group created a scalable isolation method to produce exosomes of good manufacturing practice (GMP) grade from MSCs produced from bone marrow for therapeutic applications [62]. Furthermore, therapeutic siRNA targeting oncogenic Kirsten rat sarcoma virus (KRAS) can be delivered via GMP-grade MSC exosomes. The tumor size and metastasis level in the tumor-bearing mice model were considerably reduced [63].

### Red blood cell-derived exosomes

Exosomes essentially contain a wide variety of biological components, including lipids, proteins, and nucleic acids, that are specific to their parent cells. Exosomes that have been separated from bodily fluids such as blood plasma, urine, and amniotic fluids have thus been used in the diagnosis of several illnesses [64]. Exosomes generated from red blood cells, one of these several bodily fluids, have been utilized to deliver treatments based on nucleic acids. They were proposed as a flexible delivery vehicle for therapeutic RNAs in the Le group’s prior investigation. Exosomes generated from red blood cells offer many benefits for use in clinical settings. In a nutshell, blood units, which are the source of exosomes, are easily accessible from blood banks and patients as needed. Because each blood unit contains a comparatively large volume of red blood cells (~ 1012 cells/L), there is less chance of unanticipated in vitro mutations during cell cultivation. RBCs are enucleated cell types, as opposed to other cell types that have a nucleus. This implies that exosomes that have been separated from red blood cells are not susceptible to dangers associated with genes, such as horizontal gene transfer. RBC-derived exosomes can be made to avoid triggering harmful and immunogenic reactions, just like blood transfusions can. Through the matching of donors’ and recipients’ blood types. A better transfection efficiency is offered by RBC exosomes [65].

## Food-derived exosomes

Apart from the customary constraints of exosomes produced from cells, such as low yield and potential for triggering immunogenicity [66], the restricted administration method is proposed as an area for enhancement. In light of these advantages, food such as milk and edible plants is suggested as a source of exosomes for use in clinical settings [65].

### Exosomes derived from milk

Milk is a sort of bodily fluid that adults as well as babies drink because it includes numerous elements that stimulate growth. Specifically, milk derived from cows is looking more and more like a viable alternative to exosome sources because it can be produced in large. The primary benefit of exosomes generated from milk is that, because of their durability in low pH stomach circumstances, they can effectively transfer medicinal compounds that have been encapsulated through the mouth canal. Through binding in the upper gastrointestinal tract, exosomes generated from bovine milk can induce cross-species transit via the conserved IgG-neonatal Fc receptor (FcRn). According to Agrawal et al., oral administration of paclitaxel-loaded exosomes (ExoPAC) significantly reduced tumor growth in the tumor-bearing animal model while having no negative impacts on immune responses or systemic toxicity [67]. Apart from biocompatibility and safety considerations, post-isolation modification can also be used to functionalize milk exosomes. Using polyethylene glycol (PEG), a research team under the direction of Bajpayee et al. has modified milk exosomes to enhance their integrity in acidic gastrointestinal conditions. As predicted, the mucus permeability of PEG-modified milk exosomes was around 3.2 times higher than that of untreated milk exosomes [68].

### Edible plant-derived exosomes

Plant-derived exosomes (PDEs) are also regarded as prospective options as an exosome source because they are obtained from plants and can be modified with functional moieties like folate [69]. Exosomes extracted from plants can also be delivered orally. First, it has been documented that PDEs in and of themselves protect against inflammatory diseases. Ju et al., for example, discovered that grape exosomes can regulate intestinal homeostasis and provide protection against colitis produced by dextran sulfate sodium (DSS) when taken orally [67]. Even though the in vivo experiments in this study were carried out via intravenous injection, given PDE’s therapeutic properties following oral administration [69].

## Isolation of exosomes

It is essential to separate exosomes from interfering chemicals and fragments of cells to achieve ultrapure exosomes. Exosomes can be isolated using a variety of methods, such as polymer precipitation, size exclusion chromatography (SEC), filtration, and differential centrifugation [70]. The methodologies, mechanisms, benefits, and drawbacks of exosome isolation techniques are displayed in Table 4 [71].

**Table 4:** Isolation methods of exosomes

Isolation methods	Principle	Advantages	Disadvantages	References	
Size exclusion liquid chromatography	Separation is based on size using porous polymeric beads; large molecules elute first while smaller ones enter pores	Preserves exosome integrity; separates exosomes from lipoprotein and protein contaminants	Limited throughput; lengthy process	[76]	
Density Gradient Ultracentrifugation	Separates components based on differences in density within a sucrose or iodixanol gradient	High purity; preserves vesicular structure	Time-consuming; requires expensive equipment	[74]	
Ultracentrifugation	Uses centrifugal force to isolate exosomes based on size, density, and shape	Capable of producing large quantities; widely used	Potential structural damage to exosomes; contamination with protein aggregates or microvesicles	[71, 72]	
Ultrafiltration	Filters exosomes using membranes with defined molecular weight cutoffs or pore sizes	Faster and simpler than ultracentrifugation; suitable for large-scale isolation	Risk of membrane fouling; limited size-specific separation	[72]	
Immunoaffinity capture-based techniques	Uses antibodies immobilized on beads or surfaces to capture specific exosome surface markers	High specificity for target exosomes; effective for low-abundance samples	Expensive; not suitable for bulk isolation	[79, 80]	
Microfluidic technologies	Combines physical (size, density) and biological (surface marker) properties for exosome isolation	High sensitivity; allows high-throughput analysis and automation	Requires specialized equipment; limited scalability	[72, 81, 82]	
Precipitation technique	Utilizes polymers (e.g., polyethylene glycol) to reduce exosome solubility and precipitate vesicles	Simple, inexpensive, and scalable	Co-precipitate non-exosome contaminants	[77, 78]	

### Ultracentrifugation

It is said that the traditional and gold-standard technique for separating exosomes is ultracentrifugation. Centrifugal force is used in this technique to condition biological fluids or cell culture conditions to remove big cell debris and cells based on their size, density, and form [72]. The following is an experimental methodology for ultracentrifugation described by Théry et al. to collect and isolate exosomes: To extract or separate the live cells, the culture-conditioned media is first centrifuged at 300×*g* for 10 min. After the collected supernatant has been centrifuged for 10 min at a centrifugation force of 2000×*g* to precipitate the dead cells, the supernatant is centrifuged for 30 min at 10,000×*g* to remove cell debris and then for 70 min at 100,000×*g* to precipitate the exosomes. To get rid of the tainted proteins, a lot of phosphate-buffered saline (PBS) is used to wash the collected pellet. The resulting solution is centrifuged at 100,000×*g* for 70 min. The effects of centrifugal force on exosomes can cause the exosome membrane to rupture, even though ultracentrifugation is a well-established technique for isolating exosomes [73].

### Density gradient ultracentrifugation

The foundation of this technique is a rise in the solution density gradient from the tube’s top to bottom. Contaminants whose densities differ from that of exosomes will be layered after centrifugation, and exosomes will settle into other layers that correspond to their density. The most widely utilized gradient media for exosome isolation are sucrose, iodixanol in water, and ice-cold PBS. A theoretically pure fraction of exosomes can be produced by separating impurities whose density differs from that of the exosomes using the gradient density of sucrose. When compared to other physical exosome isolation techniques, density gradient ultracentrifugation is thought to be among the best because of its purity, yield, and ability to preserve vesicular structure. Nonetheless, it has been noted that low-density lipoprotein (LDL) and high-density lipoprotein (HDL) might contaminate the exosome-containing fraction. The ultracentrifugation-derived exosomes have the potential to disrupt non-exosome microvesicles such as apoptotic bodies and protein aggregates. Ultracentrifugation with a density gradient can be used to get around this [74].

### Ultrafiltration

Standard membrane filters with specified molecular weight or size exclusion criteria can be used to separate exosomes from other extracellular vesicles, soluble protein aggregates, and cell detritus. Exosomes can be separated based on their size because they are tiny. Ultrafiltration is commonly employed as the last stage in chromatography and as a step after ultracentrifugation. The following is the standard ultrafiltration protocol: Tangential flow filtration (TFF) of the filtrate through a filter with a molecular weight cutoff of 500 kDa at a process temperature of 4 °C; dead-end filtering with a 0.1 mm filter at 22 °C to extract floating cells and cell wastes from the cell supernatant of cells; additional filtration of the deposits from step 2 using a sterilized 100 nm filter. Direct flow filtration or tangential flow filtration can be used for ultrafiltration. Nevertheless, it has drawbacks, including membrane fouling and poor particle separation. Large-scale exosome isolation can be accomplished more quickly, easily, and efficiently with tangential flow filtration also referred to as crossflow filtering. To prevent clogging or cake formation, the sample fluid in TFF travels tangentially across the filter membrane. To remove impurities smaller than 500 kDa, the retentate is further serially reconcentrated using TFF [75]. The purified exosomes are kept at 80 °C in 0.1 M sucrose. After ultrafiltration is used for isolation, it can be supplemented with additional methods like chromatography to improve the quality of the exosomes.

### Size exclusion chromatography (SEC)

Depending on the size of the exosome, big molecules and other particulate materials are separated in SEC by using a porous stationary phase. The sample of interest’s small hydrodynamic radius components can pass through the pores, causing late elution. On the other hand, early elution results from components (exosomes) having a comparatively higher hydrodynamic radius finding it difficult to enter the pores. Transmission electron microscopy has verified reports that exosomes isolated from the SEC can retain their exosome structure. Moreover, unlike the centrifugal approach, shear force won’t compromise the structure and integrity of the exosomes after their separation from the SEC [76]. This is because SEC may be carried out at low pressure, preserving exosome integrity while they are isolated. This method mostly aids in the extraction of lipoprotein or protein contaminants from the extracted exosomes. This procedure has also been applied as an ultrafiltration and ultracentrifugation method’s subsequent isolation technique. However, the SEC technique’s applicability for sample isolation is limited by its comparatively long operating time.

### Precipitation technique

This method uses hydrophilic polymers, including polyethylene glycol (PEG), to precipitate exosomes based on charge. By stealing the water molecules and forcing exosomes, PEG reduces the solubility of exosomes. In a nutshell, exosomes precipitate when exosome-containing samples are co-incubated with a PEG solution (molecular weight: 8000 Da). After being incubated at 4 °C for an entire night. This approach is comparatively simple to use and does not take a lot of running time [77]. It also does not require specific equipment. According to Kanchi et al., the polymeric networks of Tamm-Horsfall protein can be removed or separated by employing a dl-dithiothreitol solution to isolate exosomes from urine fluid using the precipitation approach. Exosomes were then precipitated at 25 °C for 30 min using a 10,000 centrifugal force. Exosomes separated using the precipitation approach are more easily recovered and resuspended than those isolated using the ultracentrifugation method [78].

### Immuno-isolation

Using magnetic beads covered with antibodies, the immune-isolation or immunoaffinity approach distinguishes between certain proteins on the lipid bilayer membrane of exosomes and other substances. According to reports, exosomes from tumors, human exosomes, and acute myeloid leukemia blasts are frequently identified using biomarkers like CD34, CD63, and CD326. Exosomes can be isolated using an immunoaffinity isolation kit on the tetraspanin proteins and exosome surface indicators, which are thought to be determining factors for the immune isolation technique [79]. The immune-isolation approach is better at selectively catching small amounts of plasma than ultracentrifugation. As a result, it is frequently employed to further isolate the particular Exosomes that have already been separated using alternative methods. However, this methodology can be employed for the extraction of exosomes specific to the individual biomarkers [80].

### Chip isolation techniques derived from microfluidics

Microfluidics-based chip isolation approaches have emerged as a viable method for exosome separation in recent years [81]. These techniques rely on the distinctions between the exosomes’ physical and biological characteristics, including their size, density, and immunoaffinity. Three methodologies may be distinguished between the purification and separation processes that use microfluidics-based chip isolation techniques: the immunoaffinity-based exosome trapping strategy, the sieving approach, and the exosomes being adsorbed into the porous structure approach [72]. The sample preparation processes for all three approaches require off-chip operations, which increase processing complexity. This method selectively entraps exosomes with a size range of 40–100 nm, and the exosomes have high selectivity, especially when used in conjunction with the microfluidic-chip-based immunoaffinity capture method. The clinical market entrance of this technology may be restricted, nonetheless, by the need to economically produce and efficiently separate the exosomes in large enough quantities [82].

## Promising strategies for high exosome scalability

The intrinsic complexities of exosome production gift hurdles that prevent smooth scaling. The problematic nature of cell processes concerned with exosome biogenesis, coupled with the want for excessive purity and yield, demands tailored solutions to propel exosome manufacturing performance to new heights. A comprehensive, multifaceted approach is needed to address the scaling issues.

### Pretreating manipulations

Pretreat parent cells is stimulating exosome production through different environmental stimulators or medium composition, such as pH, oxidative stress, and glucose starvation [83].

### Hypoxia and oxygen stress

Several studies have described the fascinating phenomenon whereby mesenchymal stem cells (MSCs) secrete more exosomes and enhance the therapeutic effects of exosomes in hypoxic environments. Hypoxia-treated MSC-derived exosomes (MSC-EXO) demonstrated greater amelioration of myocardial infarction compared to untreated MSC-EXOs, according to studies conducted in an infarcted heart model. This was demonstrated by reduced myocardial apoptosis, reduced fibrosis, and increased vascular density. The elevation of vascular endothelial growth factor (VEGF) expression and the promotion of angiogenesis were also linked to the hypoxic environment [84].

Additionally, after ethanol-induced stimulation of retinal pigment epithelial cells, oxidative stress promotes the production of tiny extracellular vehicles (EVs). Notably, these circumstances were linked to an increase in the released EVs’ vascular endothelial growth factor receptor mRNA cargo [85]. The interaction between inflammation and the tumor microenvironment was highlighted as a significant component influencing the kinetics of EV release, in addition to hypoxia and oxidative stress. Dendritic cells treated with Interferon-γ (IFN-γ) showed increased production of exosomes and tiny EVs in the setting of inflammation. These exosomes were enriched with miRNAs linked to oligodendrocyte re-myelination. Furthermore, a large body of data indicates that tumor cells coordinate significant extracellular vesicle secretion, in which these vesicles carry a variety of protein cargos and tumor-specific antigens, therefore promoting tumor growth [86].

### Calcium chloride (CaCl_2_)

Calcium (Ca^2+^) stands out as a prominently researched element known to influence the release of extracellular vehicles [87]. The effects of intracellular and external Ca^2+^ concentrations on exosome secretion in various biological settings have been the subject of several studies. More specifically, research investigating the incubation of glioblastoma cells with increasing CaCl_2_ concentrations found that the emission of EVs from the tumor increased in tandem, highlighting the stimulatory effect of elevated extracellular CaCl_2_ levels [88].

### Cytokines

In contemporary biomedical research, the utilization of cytokine pretreatment has emerged as a standard practice to augment both the yield and therapeutic efficacy of exosomes, thereby holding significant promise in the realm of brain cancer therapeutics. Interestingly, a comparison of untreated MSC-derived exosomes and IFN-γ-pretreated MSC-EXO revealed that the latter had better effects in preventing peripheral blood mononuclear cell proliferation, perhaps due to the action of indoleamine 2, 3-dioxygenase (IDO). Alongside this improvement, pro-inflammatory cytokines were decreased, Treg induction was facilitated in vitro, and neuroinflammation and demyelination in experimental autoimmune encephalomyelitis (EAE) mice were lessened. Furthermore, several studies highlight how cytokine-induced changes in exosome payloads might improve treatment results in the setting of brain cancer [89]. Investigating how IL-1β pretreatment affected BMSC-derived exosomes (BMSC-EXO) showed that miR-146a expression was upregulated, which caused macrophages to differentiate into an anti-inflammatory M2 phenotype and therefore reduce sepsis. Likewise, in osteoarthritis SW982 cells, IL-1β-primed MSC-EXOs demonstrated enhanced anti-inflammatory capabilities, mostly due to miRNAs such as miR-147b that functioned by blocking the Nuclear factor kappa β (NF-κβ) pathway [90].

### Genetic manipulation

The total amount of exosomes produced can be greatly impacted by altering important genes involved in exosome synthesis and recycling. The primary issue with this approach is the degree to which the exosomes generated in the modified routes differ from those generated in the non-manipulated pathways; more investigation is required to elucidate these distinctions. Additionally, some scientists hypothesize that the biological roles of these recently generated exosomes are comparable to those of natural exosomes. To influence cellular exosome secretion, genetic engineering approaches target important genes involved in exosome biosynthesis (e.g., Signal-transducing adaptor molecule (STAM1), Tumor susceptibility gene 101 (TSG101), CD63, CD82, CD9, hepatocyte growth factor-regulated substrate (Hrs), and HSP70) and release [e.g., the Rab family, Vesicle Associated Membrane Protein 7 (VAMP7), VAMP8, Syntaxin-5 (STX5), and synaptosome-associated protein-23 (SNAP23)]. The goal is to comprehend the distinctions between natural and genetically modified exosomes. Using certain genes such as six‐transmembrane epithelial antigen of the prostate 3 (STEAP3), syndecan-4, and L-aspartate oxidase, Kojima et al. developed the EXO device for effective exosome manufacturing, increasing output by 40 times without changing the size of the exosomes [91].

### 3D-Culturing

Emerging evidence indicates that 3D-derived exosomes (3D-EXO) exhibit higher abundance and activity compared to 2D-derived exosomes (2D-EXO), leading to enhanced therapeutic effects By supporting the paracrine actions of mesenchymal stem cells and increasing macrophage recruitment and polarization towards a healing-enhancing phenotype, the use of macroporous or fibrous scaffolds can eventually advance tissue regeneration and repair [92]. Notably, 3D-EXO was produced at a rate that was 7.5 times greater than 2D-EXO by cultivating cells in a hollow-fiber bioreactor. Additionally, 3D-EXO showed improved efficacy in encouraging cartilage regeneration. Additionally, 3D-EXO has demonstrated improved results in several applications, such as improving memory and cognitive impairments in mice with Alzheimer’s disease, protecting the kidneys, increasing the osteogenic capacity of BMSCs, promoting cell migration and proliferation, and preventing apoptosis. The distinctive spherical structure of cells induced by 3D culture reshapes the cellular microenvironment, augmenting exosome activity and yield. Because of the restricted oxygen supply, growing BMSC in 3D spheroids causes a hypoxic condition in the center, which eventually increases exosome secretion and highlights the potential of 3D culture settings for improving exosome functioning [93].

### Utilization of microfluidic devices

The integration of microfluidics offers a promising solution to address these challenges by enabling streamlined isolation and purification processes, leading to enhanced scalability and operational efficiency [94]. Utilizing a double filtration microfluidic device that leverages size exclusion principles allows for rapid isolation of exosomes in point-of-care (POC) settings. This innovative device can effectively extract exosomes from 50 to 100 μL of plasma within a mere 50-min timespan [80]. In a comparative study with the conventional polyethylene glycol-based precipitation method, the microfluidic device demonstrated comparable exosome sizes and purity levels. However, notably, exosomes isolated through the microfluidic device exhibited an earlier miRNA detection compared to those obtained through the PEG-based isolation technique. This underscores the potential of microfluidics in expediting exosome analysis and underscores its efficacy in enhancing biomarker detection speed and accuracy [95].

## Exosomes drug loading techniques

Exosome acts as a natural defense against cargo destruction during blood circulation. However, medication loading into exosomes is difficult due to its endogenous composition and lipid bilayers. In general, the medicine can be sorted into exosomes using both passive and active loading techniques (Table 5) [96]. Active loading, sometimes referred to as remote or post-drug loading, involves incubating the medication with separate exosomes. Exosomes sorted by drug are secreted from donor or source cells that have been pretreated. This process is known as passive drug loading, or the preloading approach. Adding medication to the exosome vesicle is not necessary with this technique. Because of its active pumping mechanisms, the active loading technique is more successful in achieving a greater drug/vesicle ratio. Hydrophobic medicines respond better to the post-loading strategy than hydrophilic ones [97].

**Table 5:** Exosomes drug loading techniques

Type	Drug loading methods	Advantage	Disadvantage	Principe	References	
Passive loading	Incubation of exosomes and free drugs Incubation of tumor cells with free drugs	Straight forward process. Does not jeopardize the integrity of the membrane	Loading effectiveness. Medications may be cytotoxic	Cargo diffusion across an exosomal membrane or inside a cell	[98, 99]	
Passive loading	Sonication	Greater loading capacity compared to the basic incubation technique	Sonication-induced membrane damage is a roadblock for large-scale applications	Formation of micropores using physical shear stress for diffusion	[100]	
Active loading	Extrusion	High effectiveness of freight loading. Consistent extrusion results in a uniform mixture of cargo-loaded exosomes	Exosomes’ immune-privileged status may be jeopardized by recombination of their surface structure, which would reveal exosomes to immune cells	Membrane recombination	[101, 102]	
Active loading	Freeze–thaw cycles	Easy and efficient method for directly loading several cargos into exosomes	Exosome accumulation and protein degradation may result from repeated cycles of freezing and thawing	Membrane fusion	[103]	
Active loading	Electroporation	High effectiveness of packing	The principal constraints include loading efficiency and baggage aggregation	Formation of micropores for the electrical field’s dispersion	[104, 105]	
Active loading	Membrane permeabilizers	Greater loading capacity when compared to the straightforward incubation technique	Because saponin is hemolytically active in vivo, its toxicity at higher concentrations, when utilized for drug loading, is constrained	Dissolves cholesterol-containing membrane molecules and leaves pores on the surface of exosomes	[106, 107]	

### Passive loading method

#### Incubate exosome with drug

**Fig. 8:**
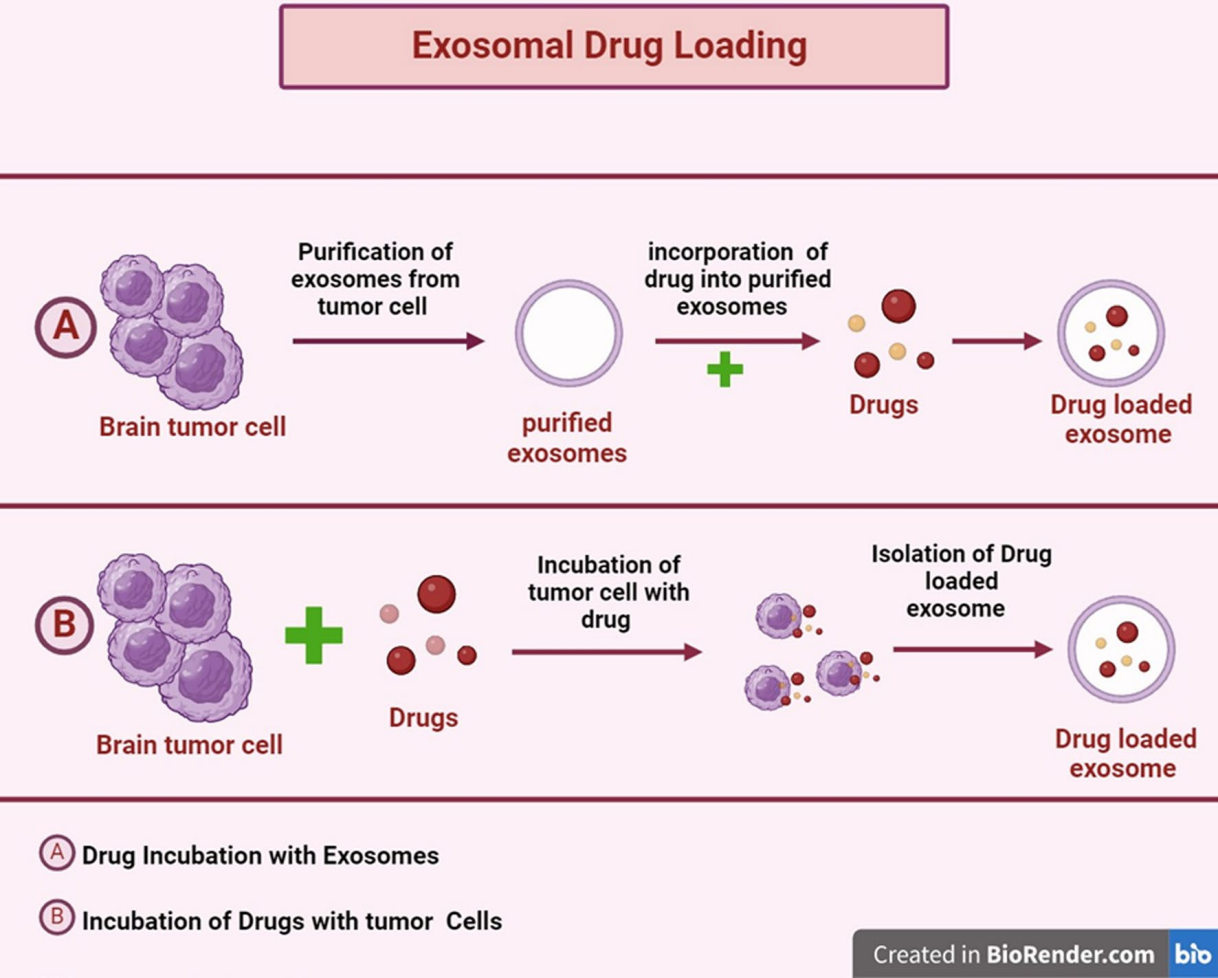
Exosomal drug loading by passive method

In the passive drug incorporation technique, the drug and exosomes are incubated together. Because hydrophobic pharmaceuticals can interact with the lipid bilayer, the efficiency achieved with this approach is directly correlated with the hydrophobicity of the drug molecules. In one investigation, Dongmel et al. incubated curcumin-treated mouse lymphoma-derived exosomes in PBS for five minutes at 22 °C. The mixture was subsequently centrifuged using a different sucrose gradient. When compared to free curcumin, the solubility, stability, and bioavailability of curcumin were increased upon encapsulation into exosomes. Similar to this, Vashisht et al. found that a loading efficiency of 70.46% was obtained when curcumin was incubated with exosomes [98] (Fig. 8).

#### Incubation of drugs with donor cells

This method involves treating the targeted exosome donor cells with an interesting pharmacological molecule, after which the pretreatment cells release exosomes that contain the drug. Using this method, the goal for decent cells is to gather medicinal or bioactive substances and release exosomes that can hold those substances. Because this technique is not targeted, the yield of exosomes may be minimal. Pascucci et al. administered and cultured SR4987 mesenchymal stromal cells for a full day at a low dose of paclitaxel. Following that, the cells were cleaned and reseeded in a brand-new flask with fresh media [99]. The paclitaxel-loaded exosomes were separated and extracted from the cell-conditioned media during a 48-h culture period (Fig. 8).

### Active drug loading approaches

To facilitate the easy diffusion of active cargo into the vesicles, active drug loading entails momentarily disrupting the exosome membrane. Extrusion, freeze–thaw cycles, and sonication are some of the methods utilized to damage the exosomes’ membranes. The active drug loading strategy enhanced the drug loading capacity by up to 11 times when compared to passive drug loading. The primary issue with this strategy is that during the membrane disruption process, it may harm exosomes’ original structure and target characteristics.

#### Sonication

A medicine or protein of interest is combined with exosomes produced from donor or target cells, and the mixture is sonicated using a homogenizer probe. The integrity of the exosome membrane is perturbed by the shearing force produced during sonication, which also deforms the membrane and permits the diffusion of bioactive substances into the exosome. According to Kim et al., sonication dramatically reduces the exosome membrane’s micro-viscosity. Nevertheless, this membrane deformation mechanism does not affect the lipid contents or membrane-bound proteins of the exosome. It was discovered that when incubated at 37 °C, the exosome’s membrane integrity can be restored in less than an hour. Furthermore, when the medications are contained within the exosomes and also adhered to the outer membrane layer of the exosome vesicle, biphasic drug release is occasionally seen from the exosomes [100]. Drugs that are enclosed inside exosomes release slowly, while drugs that are connected to the outside layer of exosomes explode explosively.

#### Extrusion

Extrusion is a post-loading technique that loads drugs using a lipid extruder based on a syringe. After being separated from the donor cells, exosomes are combined with a specific medication and put into a lipid extruder that is syringe-based and has a porous membrane (100–400 nm) at a temperature that is controlled. The medication is thoroughly combined with the damaged exosome membrane during the extrusion process. The benefits of the extrusion technique for drug-loading exosomes were documented by Fuhrmann et al. Porphyrin was loaded into exosomes made from MDA-MB231 breast cancer cells utilizing the extrusion technique. The cytotoxic effect of extrusion loading was higher than that of the incubation approach [101]. Moreover, the zeta potential of the initial exosomes is changed by the extrusion process, and the modification of the vesicle constitution is brought about by a higher number of extrusions which can aid in the efficiency [102].

#### Freeze–thaw cycles

When utilizing the freeze–thaw method for drug loading, exosomes are incubated with a specific medication at room temperature for a predetermined duration before being quickly frozen at – 80 °C or in liquid nitrogen. After that, the mixture is left to thaw at the ambient temperature. For the best drug encapsulation, cycles of freeze–thaw should be performed a minimum of 3 times. Comparing this procedure to sonication techniques reveals a reduced drug-loading capacity [103].

#### Electroporation

Using an electric field during the electroporation process causes the phospholipid bilayer of the exosomes to rupture, allowing drug molecules to enter the lumen of the exosomes and form holes more easily [104]. Drug molecules permeate via the pores that are created on the lipid bilayer membrane of the exosome after electroporation; in the meantime, the membrane’s integrity is restored following loading. This technique is frequently used to load big molecules into exosomes, including nucleotides (siRNA or miRNA). The limited loading capacity of the electroporation technique can be attributed to problems with exosome instability and RNA aggregation [105].

### Incubation with membrane permeabilizers

Exosomal membrane permeability can result from interactions between membrane permeabilizers and surfactants like saponin, which can generate pores. When compared to the incubation approach, the membrane permeability method can improve the catalase loading efficiency into exosomes [106]. A previous study showed that the use of saponin increased the loading of hydrophilic substances into exosomes by 11-fold compared to the inactive loading approach without saponin. This method should be used to isolate exosomes after saponin incubation and to determine the optimal amount of saponin for drug addition [107].

### Cancer treatment via exosome cargo loading

#### Anti-cancer drugs

It has been observed that both hydrophilic and hydrophobic chemotherapeutic medications, such as paclitaxel (PTX) and Dox, are loaded into exosomes. A growing body of research has demonstrated that chemotherapeutic administration via exosomes can augment anti-cancer effects. One of the best anti-cancer medications, Dox, is used to treat lymphoma, solid tumors of various kinds, and leukemia. However, because of its low biocompatibility and severe side effects such as suppressing bone marrow function and cardiac toxicity, the therapeutic usage of Dox is highly limited. While numerous attempts are being made to improve Dox’s biocompatibility and anti-cancer properties using different nanoparticle technologies, there are certain adverse effects related to nanoparticles that need to be addressed, namely oxidative stress and immunological response [108, 109]. Dox has been thoroughly investigated in exosome-mediated anti-cancer therapy because of its inherent fluorescence, which makes it easy to follow. In the colon adenocarcinoma mouse model, exosomes produced by serial extrusion from macrophages pretreated with doxorubicin exhibit stronger anti-cancer effects than groups of liposomes loaded with Dox or free Dox. Exosomes have a far higher capacity to target cancer cells than liposomes because of their enhanced method of endocytosis by cholesterol and the phospholipid makeup of their membranes [110]. Dox-loaded exosomes lessen cardiotoxicity, a common side effect of Dox, by preventing Dox from reaching cardiac endothelial cells. More recently, it was discovered that the anticancer impact of Dox in osteosarcoma may be improved by exosomes made from mesenchymal stem cells. This could be attributed to mesenchymal stem cells’ preference for tumor tissues, highlighting the significance of choosing exosome sources carefully. Another popular anti-mitotic medication for cancerous tumors like glioblastoma multiforme and breast cancer is PTX. When a patient develops cisplatin resistance, PTX is frequently utilized to overcome medication resistance. PTX’s poor bioavailability and dose-dependent toxic impact, however, pose a significant challenge to its clinical utilization. Additionally, According to multiple investigations, PTX was unable to cross the BBB. Strong anti-cancer effects were demonstrated by PTX-loaded exosomes, which were produced by mesenchymal stromal cells that had been pretreated with PTX. Additionally, PTX-encapsulated cancer-derived exosomes may specifically target drug-resistant Cancer stem cells (CSCs), enhancing their cytotoxicity against cancer cells. MDR stands for multiple drug resistance, and it is the main challenge to effective cancer treatment. It has been demonstrated that exosomes can effectively defeat multiple drug resistance (MDR) in cancers. The P-glycoprotein drug efflux transporter could be circumvented by PTX-loaded macrophage-derived exosomes [111]. Exosomes made from U-87 MG cells can carry PTX across the blood–brain barrier and overcome MDR, improving the therapeutic efficacy against glioblastoma multiforme.

#### Nucleic acids

Utilizing nucleic acids like DNA and RNA for gene therapy is a compelling and exciting approach to treating cancer. Specifically, to control the expression of genes. Liposomes and inorganic nanoparticles are examples of nano-based delivery methods that have been designed to transport nucleic acids to tumors while shielding them from endonuclease degradation. However, obstacles to delivery efficiency, stability, and safety must be removed before these gene delivery methods may be used in real-world clinical settings [112]. These nanocomplexes are typically produced by interaction electrostatics between strongly negatively charged phosphate backbone RNA and positively charged carriers. Though it is more difficult to release short RNAs for gene control, the stability is due to charge interaction strengthening the protection for RNA. Furthermore, these nanocarriers’ cationic surface charges could be harmful. Therefore, a key component of effective short RNA distribution is striking a balance between their release and protection [113].

Exosomes have gained interest recently as gene delivery vehicles because of their special qualities that allow them to get around these challenges. Exosomes were shown to contain a large number of miRNAs that are engaged in intercellular communication, some of which have anti-cancer characteristics. It has been suggested to pre-overexpress candidate RNAs in parental cells to load desired RNAs into exosomes. To create miR-122 encapsulated exosomes, adipose tissue-derived mesenchymal stem cells were transfected with the miR-122 expression plasmid. The hepatocellular carcinoma cells’ chemosensitivity was enhanced by the miR-122-loaded exosomes, which changed genes like cyclin G1 and metalloproteinase domain-containing protein 10 [114]. Furthermore, in the xenograft mouse model, intratumorally injection of these miR-122-loaded exosomes demonstrated enhanced anticancer effects.

#### Proteins

Using exosomes is the most promising method for delivering proteins. Exosomes can be directly loaded with proteins by physical loading techniques like electroporation, or they can be created by genetically modifying donor cells. An interest protein’s gene is transfected into donor cells. Consequently, the inserted genes cause the cell to produce proteins that are then secreted into exosomes. Many cancer cells depend on the anti-apoptotic protein survivin for their continued survival. This survivin is inhibited by the dormant mutant survivin-T34A, which causes cancer cells’ mitochondrial apoptotic cascade to begin. It was shown that in several adenocarcinoma of the pancreas cell lines. Because of their advantage in transporting bioactive compounds and their physiological significance in the immune system, exosomes offer significant potential as vaccine vectors [115]. Dendritic cell-derived exosomes (DEX) are one immune system-derived exosome that can elicit immunological responses like that of parental DCs. Tissue-specific antigens, peptides, and immune stimulants that can trigger the host immune system’s attack on tumor cells have recently been tried to be loaded into DEX. A mouse DC cell line was infected with a lentivirus encoding the AFP gene to employ the fetal liver protein-fetoprotein (AFP) as a hepatocellular cancer antigen [116]. Tyrosinase-related protein-2 (TRP2) was electroporated or loaded into serum-derived Exosomes using a detergent like saponin to improve membrane permeability. Immunotherapy can be used to treat cancer by using fluorescently labeled exosomes that demonstrate high signals in lymph nodes and efficiently absorb TRP2-containing exosomes into macrophages.

## Potential therapeutic strategies for treatment

### Exosomes for stimulating immune response

**Fig. 9:**
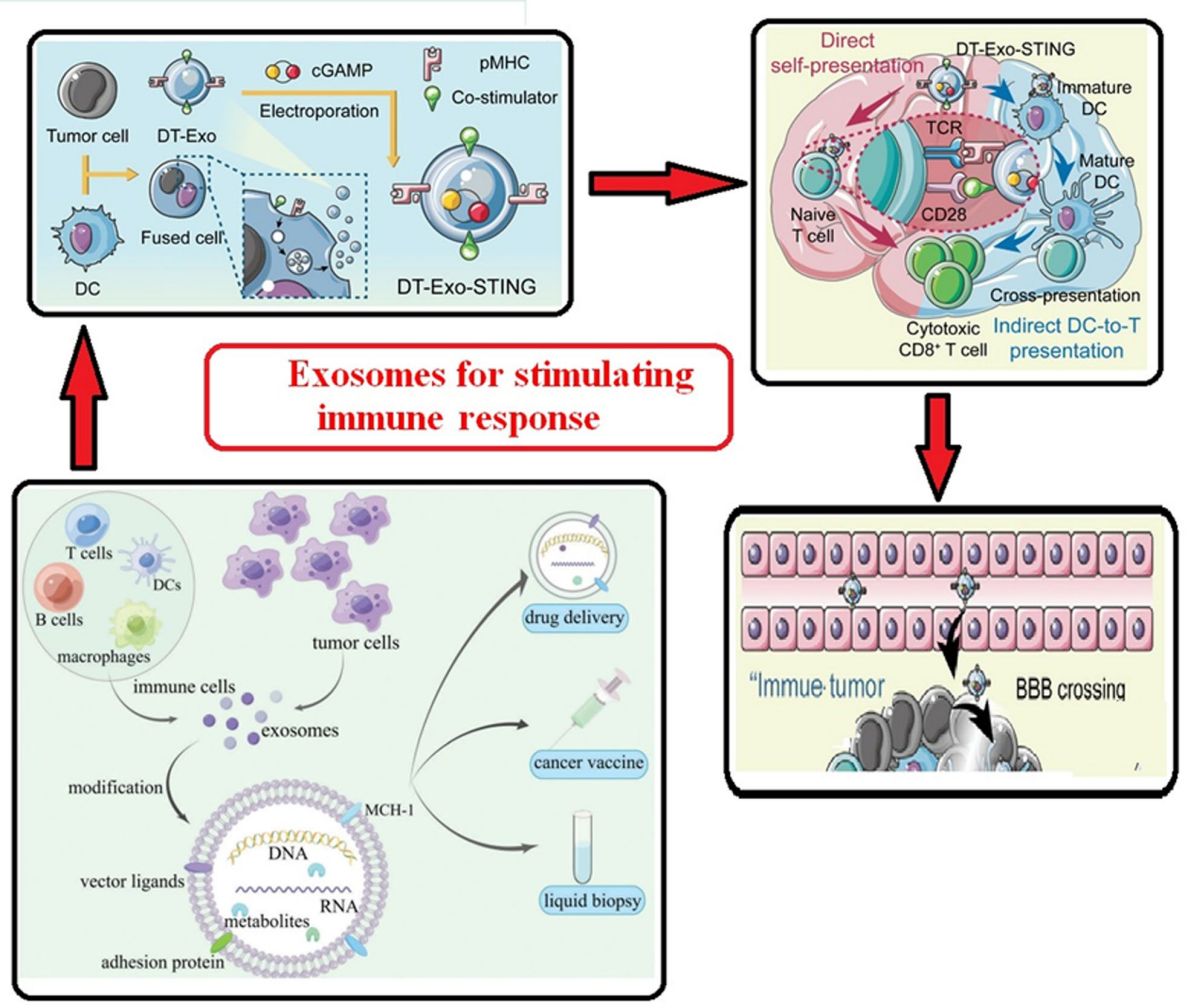
DC-tumor hybrid cell-derived chimeric exosomes loaded with STING agonists (DT-Exo-STING) delivery system that is intended to increase the defense system’s T-cell attack against cancer cells

An innovative immunotherapy approach involving the engineering of exosomes generated from a fusion between dendritic cells and tumor cells has been crafted. Stimulators of Interferon Genes (STNG) ligands are the substances that these specialized exosomes are packed with. DT-Exo-STING is a delivery system that is intended to increase the defense system’s T-cell attack against cancer cells in particular (Fig. 9) [117].

This immunotherapeutic approach is particularly novel when it comes to brain cancer. Traditional cancer treatments like radiation or chemotherapy can cause serious collateral harm to the healthy brain because of the delicate and intricate nature of brain tissue. With precisely engineered chimeric exosomes that activate T-cell responses with little effect on surrounding brain cells, DT-Exo-STING offers a glimmer of hope by enabling the activation of tumor-specific antigens CD8+ T cell lymphocytes that trigger an effective immune reaction against cancer cells [118]. The personalized DT-Exo-STING nano vaccine was used in conjunction with immune checkpoint inhibitor therapy in a preclinical investigation that used a mouse model of brain cancer to avoid post-operative glioblastoma recurrence. The study’s results were encouraging, demonstrating that mice given the nano vaccine had better survival rates and an increased immune response [119].

Furthermore, there are two sides to the inclusion of STING agonists in these exosomes. They not only encourage the direct activation of T cells but also boost the signaling pathways that further activate the dendritic cells’ immunostimulatory functions. This two-pronged approach is essential to successfully treating tumors. Sustained investigation and clinical studies are required to improve this method, maximize its effectiveness, and guarantee patient safety [117].

### Exosomes for carrying anti-tumor drugs

**Fig. 10:**
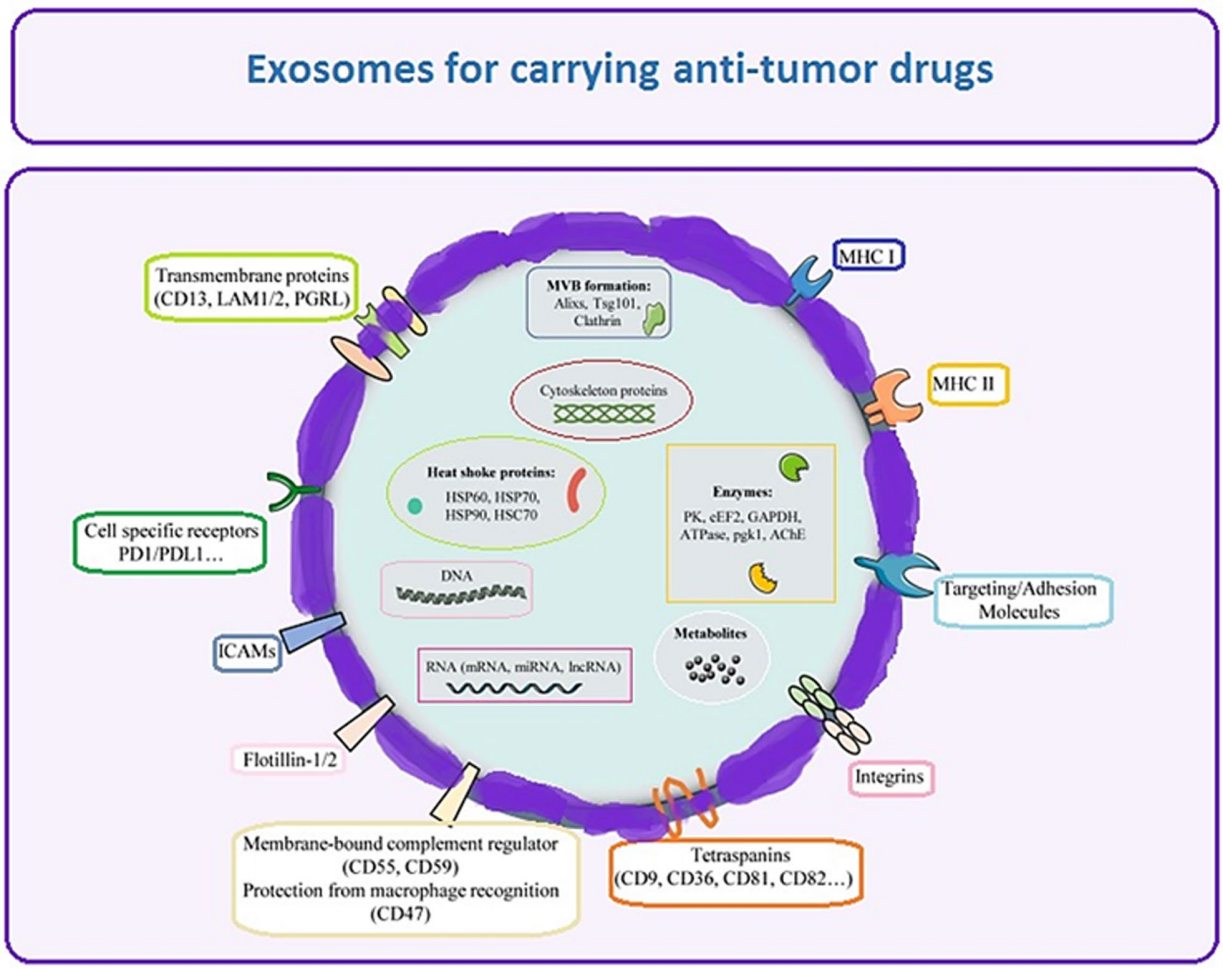
Exosomes for carrying anti-tumor drugs

Numerous immune-related chemicals that control the immune response are carried by exosomes. Tumor necrosis factor-related apoptosis-inducing ligands (TRAIL) can bind TRAIL receptors like TRAIL-R1 (DR4) and TRAIL-R2 (DR5) within tumor cells, which can trigger target cell apoptosis. TNF family proteins, such as Fas ligands, can fight brain cancer through the Fas/FasL pathway. Beyond these ligands that cause apoptosis [120], exosomes can transfer chemotherapy medications like Paclitaxel and Doxorubicin (Fig. 10) [121].

### Exosomes for carrying siRNA

Compared to the existing systemic gene therapy delivery techniques, exosome-mediated delivery shows great promise and benefits. When loaded with therapeutic nucleic acids, targeted exosomes effectively and safely reduce the rate at which tumors proliferate [122]. This tactic one possible treatment method for glioblastoma (GBM) is to target the energy metabolism of the disease by inhibiting both mitochondrial and phospholipid pathways. One novel strategy for treating GBM is to simultaneously target mitochondrial metabolism and cytoplasmic phospholipase A2 (cPLA2) by treating it with metformin and knocking down cPLA2. Blood exosomes were found to be the best vehicles for distributing this medicinal concoction. When exosomes containing metformin and cPLA2 siRNA were evaluated for cellular absorption, primary GBM cells showed encouraging therapeutic benefits. The efficacy of the exosome-mediated cPLA2 siRNA/metformin strategy and the efficiency of GBM-targeted delivery were assessed in vivo in a patient-derived xenograft (PDX) model. Both genomic analysis and experimental validation emphasized the significance of polymerase 1 and transcript release factor (PTRF) in improving exosome uptake by GBM cells, proving the feasibility of this delivery strategy [123].

### Classification as a therapeutic product

Exosomes can be classified differently depending on their origin, composition, and intended use; Biologics: Exosomes derived from cells (e.g., stem cells) are often regulated as biologics, requiring compliance with stringent guidelines, such as the FDA’s regulations under the public health service (PHS). Drugs: Exosome-based products with a defined pharmacological activity may be treated as drugs, necessitating preclinical and clinical trials. Cell or Tissue-based Products: If the exosomes are derived from human cells or tissues, additional rules may apply under the human cells, tissues, cellular, and tissue-based products (HCT/Ps) regulations. The classification determines the regulatory pathway, including preclinical and clinical requirements [124].

### Manufacturing and quality control

The production of exosome-based therapies presents challenges in ensuring consistency and quality as; source material variability: Exosomes derived from different cell types or donors may vary in composition and efficacy. Scalability: Large-scale manufacturing while maintaining product consistency is a significant challenge. Purity and safety: Rigorous methods to purify exosomes and remove contaminants (e.g., residual cells, proteins, or genetic material) are essential to minimize risks such as immune reactions or off-target effects. Characterization: There is a need for standardized methods to characterize exosomes, including size, content (RNA, proteins, lipids), and biological activity. Regulatory agencies often require detailed documentation of the manufacturing process and robust quality control measures [125].

### Preclinical studies

To demonstrate safety and efficacy, exosome-based therapies must undergo rigorous preclinical testing as; biodistribution and pharmacokinetics: Understanding how exosomes distribute in the body, their half-life, and clearance mechanisms is essential.

Toxicology: Comprehensive toxicological studies are required to assess potential risks, such as unintended immune responses or tumorigenicity. Mechanism of action: Clear elucidation of the therapeutic mechanism is often required, though this remains challenging given the complexity of exosome content [126, 127].

### Clinical trials

Exosome-based therapies face unique challenges in clinical trial design such as Patient safety: Given their novel nature, extensive safety data are needed to address potential immunogenicity or adverse effects. Dose standardization: Establishing an appropriate dosing regimen can be challenging due to variability in exosome content and activity. Endpoints: Clear, measurable clinical endpoints are necessary, particularly if exosomes are intended for regenerative medicine or as drug delivery systems [128].

### Regulatory guidance and standards

There is a lack of comprehensive regulatory frameworks specific to exosome-based therapies as; global variability: Regulatory requirements differ across regions (e.g., FDA in the U.S., EMA in Europe, and PMDA in Japan), complicating international development. Emerging Standards: Regulatory agencies are still developing guidance on exosome-based therapies. For example, defining what constitutes “substantial manipulation” or “homologous use” in the context of exosome therapies is evolving. Compliance with good manufacturing practices (GMPs): Manufacturers must meet GMP standards, which require robust documentation and validation of processes, facilities, and personnel [129].

### Ethical and legal issues

Donor material: Ethical concerns arise when exosomes are derived from human donors, including consent, traceability, and the potential for exploitation. Intellectual property (IP): Protecting IP while complying with regulatory standards can be challenging, especially for novel therapies with overlapping patents [130].

### Addressing these challenges

To facilitate clinical translation, the following strategies can help; development of regulatory guidelines: Collaboration between researchers, regulatory agencies, and industry to establish standardized guidelines and frameworks. Standardization of methods: Developing universal standards for exosome isolation, characterization, and quality control. Education and communication: Enhancing dialogue between developers and regulators to clarify expectations and resolve uncertainties early in the development process [131]. By addressing these regulatory considerations comprehensively, developers of exosome-based therapies can navigate the path to clinical application more effectively.

## Influence of patient characteristics on exosome-based therapies

Exosome-based therapies hold great promise for precision medicine, as they can be engineered to address specific pathological conditions. However, patient-specific factors, such as genetic variants and pre-existing conditions, may significantly influence the efficacy and safety of these therapies.

### Genetic variants and exosome dynamics

Patient-specific genetic variations can affect multiple aspects of exosome biology, including Exosome production: Variants in genes involved in exosome biogenesis, such as those encoding Rab GTPases and tetraspanin, may alter the quantity or quality of exosomes produced by the patient’s cells [132]. Cargo loading and composition: Genetic polymorphisms in regulatory pathways, such as those affecting miRNA or protein expression, may influence the cargo packaged into exosomes. For example, single nucleotide polymorphisms (SNPs) in miRNA-binding sites could alter the therapeutic efficacy of miRNA-loaded exosomes [133, 134]. Uptake efficiency: Variations in receptor genes, such as integrins or lectins, may affect the binding and internalization of exosomes, potentially influencing the therapeutic outcomes [135]. Genetic diseases: Exosome profiles and biological responses may be different in people with underlying medical illnesses, such as cardiovascular or neurological diseases, than in healthy people. For instance, they are linked to persistent inflammation, which can lead to the development of a pro-inflammatory milieu inside the brain. During the therapy of brain cancer, this inflammatory environment may change the behavior of exosomes, impacting their stability, cargo content, and interactions with recipient cells [133, 134]. Furthermore, genetic mutations affecting components of the endosomal-lysosomal-exosomal pathways, such as charged multivesicular body protein 6 (CHMP6), TSG101, Rab35, and Rab7A, may influence vesicle cargoes routing, Tau traffic, degradation, and secretion. These mutations could interfere with the proper sorting and trafficking of therapeutic cargoes within exosomes, thereby compromising the targeted delivery of anticancer agents to tumor cells [136].

### Impact of pre-existing conditions

Pre-existing conditions, particularly those involving inflammation or altered metabolic states, can significantly modify exosome interactions and therapeutic efficacy: Chronic inflammation: Conditions such as autoimmune diseases or cancer-associated inflammation can change the composition of circulating exosomes. These “primed” exosomes may compete with therapeutic exosomes, reducing their effectiveness [135, 137]. Diabetes and obesity: Metabolic disorders can alter the lipid and protein composition of exosomes, potentially impairing their ability to deliver therapeutic cargo efficiently. Immune status: Patients with immunodeficiencies or hyperactive immune responses may experience altered biodistribution or clearance of therapeutic exosomes [138]. Environmental contaminants: Exosomes’ protein composition can change in a variety of ways due to environmental pollutants, which might reveal how cells are responding to stress, inflammation, or unfavorable conditions. For example, exosomes carrying inflammatory cytokines including interleukins, tumor necrosis factor-alpha, and chemokines can be released in response to exposure to pollutants, especially those that have pro-inflammatory properties [137]. As a result, these proteins may intensify immunological and inflammatory reactions, and exposure to toxins that harm cells may result in the presence of molecular patterns linked to damage in exosomes. These may involve proteins that show signs of apoptosis or cell damage [138].

### Precision medicine approaches

To address these challenges, exosome-based therapies can be tailored to individual patient profiles: Genetic screening: Screening for genetic variants associated with exosome production or uptake could help predict patient responsiveness to therapy. For instance, patients with altered receptor expression may benefit from exosomes engineered to target alternative pathways [135]. Condition-specific exosomes: Developing exosomes derived from patient-specific cell types, such as mesenchymal stem cells or immune cells, may ensure better compatibility and therapeutic efficacy [136]. Adaptive engineering: Customizing exosome surface markers or cargo based on a patient’s disease state and genetic background could enhance targeting and reduce off-target effects.

## Challenges and perspectives

Because of their special characteristics, including their innate source exosomes present exciting opportunities to improve the administration of medicines. Recognizing the many benefits exosomes offer. It is vital to bear in mind that exosomes presently lack approval from the Food and Drug Administration (FDA) for the treatment or diagnosis of any disease. Consequently, their use needs to be limited to clinical research or FDA biologics licensing applications.

One significant step was taken in 2020 when the FDA released a consumer advisory warning people about the possible risks and unsupported claims related to unregulated exosome treatments. In particular, individuals in Nebraska who received unapproved medicines allegedly containing exosomes reported experiencing serious side consequences. Interestingly, no FDA-approved exosome products are currently on the market; thus, clinical studies must be planned, developed, coordinated, and carried out immediately [139]. Notwithstanding the encouraging results of clinical studies, concerns have been raised about new data showing that three clinical trials are now underway that focus on altering mesenchymal stem cells to increase the production of exosomes. However, as these studies have not yet been completed, the results are still pending publication [140].

The ongoing clinical trials (six trials registered on clinical trials.gov, accessed on 23 March 2023) on the utilization of exosomes for the delivery of medicinal substances have addressed this problem [140]. Concerning the employment of exosomes for delivering therapeutic agents, resolved or rebutted this issue. Nonetheless, it will be sufficiently tough to assess the therapeutic potential of utilization of autologous or non-autologous exosomes for the treatment of brain cancer, investigated through experiments on human brain organoids and subsequent clinical trial applications [141]. The selection of the exosome source is crucial for therapeutic applications. Exosomes generated from tumors have a remarkable ability to target cancer cells, and they carry bioactive cargo that may either directly or indirectly encourage the progression of cancer. To address the challenges posed by varied exosome subpopulations in the future, methods for identifying, removing, or adding exosomal components are essential for exosome-based drug delivery for cancer treatment with concern that the uniformity of storage conditions has an impact on exosome performance as well.

Several techniques, including sonication, transfection, electroporation, and incubation, have been developed recently to load therapeutic payloads into exosomes. But exosome-cargo-loading methods as they exist now are insufficient to meet the loading efficiency needed for clinical applications. Specifically, there are many restrictions on the kind of cargo that may be loaded using the basic incubation approach, and its low efficiency makes it unsuitable for use in clinical settings. The cost of mass production should be decreased and the process further simplified by transformation techniques. Currently, available physical treatments like electroporation are the most effective way to incorporate nucleic acids like miRNA or siRNA into exosomes. However, new strategies are required because this process has the potential to alter the characteristics of exosomes and to cause the aggregation and destruction of charged nucleic acids [142]. Their low yields are a significant barrier to the clinical utilization of exosomes. The majority of preclinical experimental research uses cell culture to produce exosomes. Less than 1 mg of exosomal protein is generated per milliliter of culture, despite some variations based on the type of donor cells. Making exosome-mimetic nanovesicles (EMNVs) may be a different tactic to get around this restriction. Extruding cells through successive micrometer-sized filtering results in EMNVs. The yield of EMNVs is improved by about 100 times using these serial extrusion procedures, and anticancer medications can be encapsulated at the same time. It is imperative to elucidate the alterations in vivo (PK/PD) due to the potential impact on the vesicle membrane composition during the cell extrusion procedure. Furthermore, research teams have created hybrid EMNVs known as exosome-liposome hybrids, which combine exosomes with artificial liposomes. In brief, the following three processes serve as examples for creating hybrid EMNVs: freeze–thaw, simple incubation, and extrusion [115].

To create hybrid EMNVs, the authors of a work by Lin et al. loaded liposomes with plasmid vectors carrying Cas9 and then treated them with exosomes loaded with sgRNA that were recovered from HET293 cells. The CRISPR/Cas9 system is delivered to MSCs by these hybrid exosome-like vesicles, which also successfully cleave the target genes. In comparison to traditional drug delivery methods, hybrid EMNVs and milk-derived exosomes offer a range of advantages. These advanced vesicles are derived from natural sources, enhancing biocompatibility and reducing the risk of immune reactions associated with synthetic nanoparticles. Moreover, they can be precisely engineered to target specific tissues or cells, enabling accurate drug delivery while minimizing off-target effects. Additionally, milk-derived exosomes contain bioactive molecules and genetic material that can modulate biological processes, providing a natural and potent approach to therapeutic intervention [135]. Additionally, several studies are attempting to circumvent the low-yield hurdle by employing exosomes derived from different diets. It makes sense that the superior cellular absorption efficiency and general safety of these food-derived Exosomes are drawing attention. Specifically, exosomes generated from milk demonstrated a yield that was 1000 times higher than those derived from animal cell cultures. Furthermore, the intestinal absorption of milk exosomes administered orally was found to be enhanced.

**Fig. 11:**
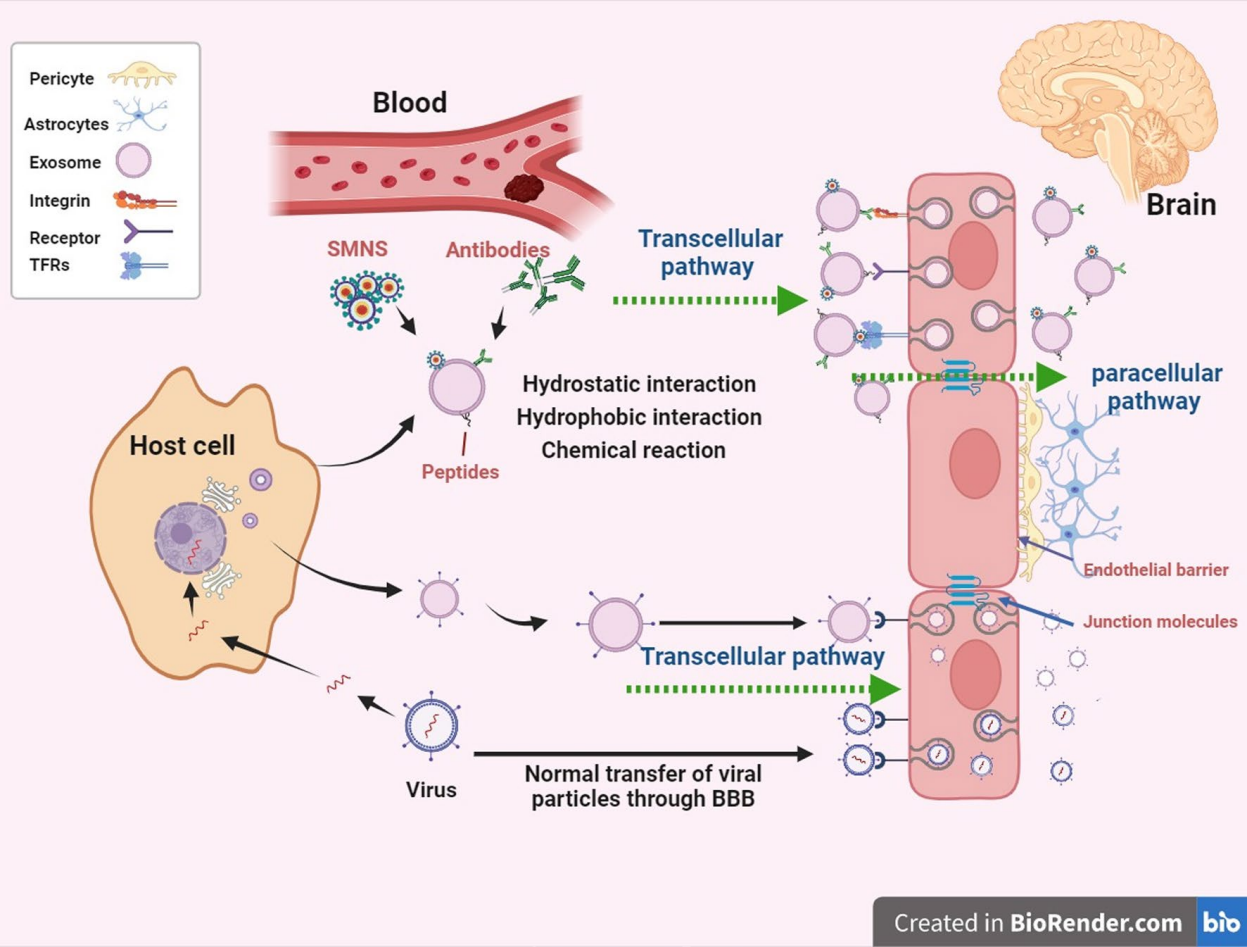
Summary of transportation of drug-loading exosomes through the blood–brain barrier (BBB)

Exosomes offer limitless potential as biomarkers for cancer detection and prognosis, in addition to their use as drug carriers [143]. To help with the clinical uses of exosomes and to investigate their diverse profiles and roles, a great deal of research has been conducted. Exosomes that are separated from diverse body fluids, like blood, saliva, and urine, have the potential to be used as cancer biomarkers since they can detect aberrant cell physiology. However, the exact identification of distinct signals that vary depending on the source presents a significant problem (Fig. 11).

## Conclusion

Exosomes represent a promising avenue for targeted drug delivery in the treatment of brain cancer. Thus, this review elucidated the potential of exosome-based therapies to revolutionize the way to approach brain cancer treatment, by leveraging exosomes as nanocarriers, there is an opportunity to maximize treatment impact while minimizing damage to healthy brain tissue. However, it is important to acknowledge the existing challenges, such as scalability and the intricate interplay between exosomes and the tumor microenvironment. Addressing these challenges will be crucial for the successful clinical translation of exosome-based therapies. Overall, the potential of exosomes in brain cancer treatment is immense, and continued research and innovation in this area holds great promise for improving patient outcomes and quality of life.

## Data Availability

No datasets were generated or analysed during the current study.

## Abbreviations

TP53: Tumor protein 53
PTEN: Phosphatase and TENsin homolog
NF1: Neurofibromatosis type 1 genes
CTCs: Circulating tumor cells
L1CAM: L1 cell adhesion molecule
STAT3: Signal transducer and activator of transcription 3
FasL: Fas ligand
BBB: Blood–brain barrier
JAMs: Junctional adhesion molecules
PI3K: Phosphatidylinositol-3 kinase
PA: Plasminogen activator
serpin: The serine proteinase inhibitor
OXPHOS: Oxidative phosphorylation
WBR: Whole brain radiation therapy
SRS: Stereo static radiosurgery
ROS: Reactive oxygen species
EPR: Enhanced permeability and retention
EE: Early endosomal
ESCRT-0: Endosomal Sorting Complex Required Transport
ILVs: Intraluminal vesicles
MVBs: Multi-vesicle bodies
HSP70, HSP90: Heat shock proteins
MHC: Integrins myosin heavy chain
mRNA: Messenger RNA
miRNA: MicroRNA
UTR: 3'-Untranslated region
THP-1: Human monocytic leukemia cell line
TRPP2: Transient receptor potential polycystic 2
NK: Natural-killer
IL-6: Interleukin 6
TNF-α: Tumor necrosis factor-alpha
TAMs: Tumor-associated macrophages
TEXs: Tumor-derived exosomes
TME: Tumor microenvironment
MSCs: Mesenchymal stem cells
DCs: Dendritic cells
DDS: Drug delivery systems
EVs: Extracellular vesicles
HEK: Human embryonic kidney
mVSVG: Minimal vesicular stomatitis virus glycoprotein
BMDMs: Bone marrow-derived macrophages
BMDCs: Bone marrow-derived dendritic cells
ECM: Extracellular matrix
Dox: Doxorubicin
Rab: Ras-associated binding
DEX: DC-derived exosomes
KRAS: Kirsten rat sarcoma virus
FcRn: IgG-neonatal Fc receptor
ExoPAC: Paclitaxel-loaded exosomes
PEG: Polyethylene glycol
PDEs: Plant-derived exosomes
DSS: Dextran sulfate sodium
PBS: Phosphate-buffered saline
LDL: Low-density lipoprotein
HDL: High-density lipoprotein
TFF: Tangential flow filtration
SEC: Size Exclusion Chromatography
MSC-EXO: Hypoxia-treated MSC-derived exosomes
VEGF: Vascular endothelial growth factor
IFN-γ: Interferon-γ
Ca^2+^: Calcium
IDO: Indoleamine 2, 3-dioxygenase
EAE: Experimental autoimmune encephalomyelitis
BMSC-EXO: BMSC-derived exosomes
NF-κβ: Nuclear factor kappa β
STAM1: Signal-transducing adaptor molecule
TSG101: Tumor susceptibility gene 101
Hrs: Hepatocyte growth factor-regulated substrate
VAMP7: Vesicle Associated Membrane Protein 7
STX5: Syntaxin-5
SNAP23: Synaptosome-associated protein-23
STEAP3: Six‐transmembrane epithelial antigen of the prostate 3
3D-EXO: 3D-derived exosomes
2D-EXO: 2D-derived exosomes
POC: Exosomes in point-of-care
PTX: Paclitaxel
MDR: Multiple drug resistance
CSCs: Cancer stem cells
AFP: Protein-fetoprotein
TRP2: Tyrosinase-related protein-2
STNG: Stimulator of Interferon Genes
TRAIL: Tumor necrosis factor-related apoptosis-inducing ligands
GBM: Glioblastoma
cPLA2: Cytoplasmic phospholipase A2
PDX: Patient-derived xenograft
PTRF: Polymerase 1, Transcript release factor
PHS: Public health service
HCT/Ps: Cellular and tissue-based products
GMPs: Compliance with good manufacturing practices
IP: Intellectual property
SNPs: Single nucleotide polymorphisms
CHMP6: Charged multivesicular body protein 6
FDA: Food and Drug Administration
EMNVs: Making exosome-mimetic nanovesicles
